# STING, the Endoplasmic Reticulum, and Mitochondria: Is Three a Crowd or a Conversation?

**DOI:** 10.3389/fimmu.2020.611347

**Published:** 2021-01-21

**Authors:** Judith A. Smith

**Affiliations:** Department of Pediatrics and Medical Microbiology and Immunology, University of Wisconsin-Madison, Madison, WI, United States

**Keywords:** STING, cGAS, mitochondria, endoplasmic reticulum, unfolded protein response, reactive oxygen species

## Abstract

The anti-viral pattern recognition receptor STING and its partnering cytosolic DNA sensor cGAS have been increasingly recognized to respond to self DNA in multiple pathologic settings including cancer and autoimmune disease. Endogenous DNA sources that trigger STING include damaged nuclear DNA in micronuclei and mitochondrial DNA (mtDNA). STING resides in the endoplasmic reticulum (ER), and particularly in the ER-mitochondria associated membranes. This unique location renders STING well poised to respond to intracellular organelle stress. Whereas the pathways linking mtDNA and STING have been addressed recently, the mechanisms governing ER stress and STING interaction remain more opaque. The ER and mitochondria share a close anatomic and functional relationship, with mutual production of, and inter-organelle communication *via* calcium and reactive oxygen species (ROS). This interdependent relationship has potential to both generate the essential ligands for STING activation and to regulate its activity. Herein, we review the interactions between STING and mitochondria, STING and ER, ER and mitochondria (vis-à-vis calcium and ROS), and the evidence for 3-way communication.

## Introduction

**Figure 1:**
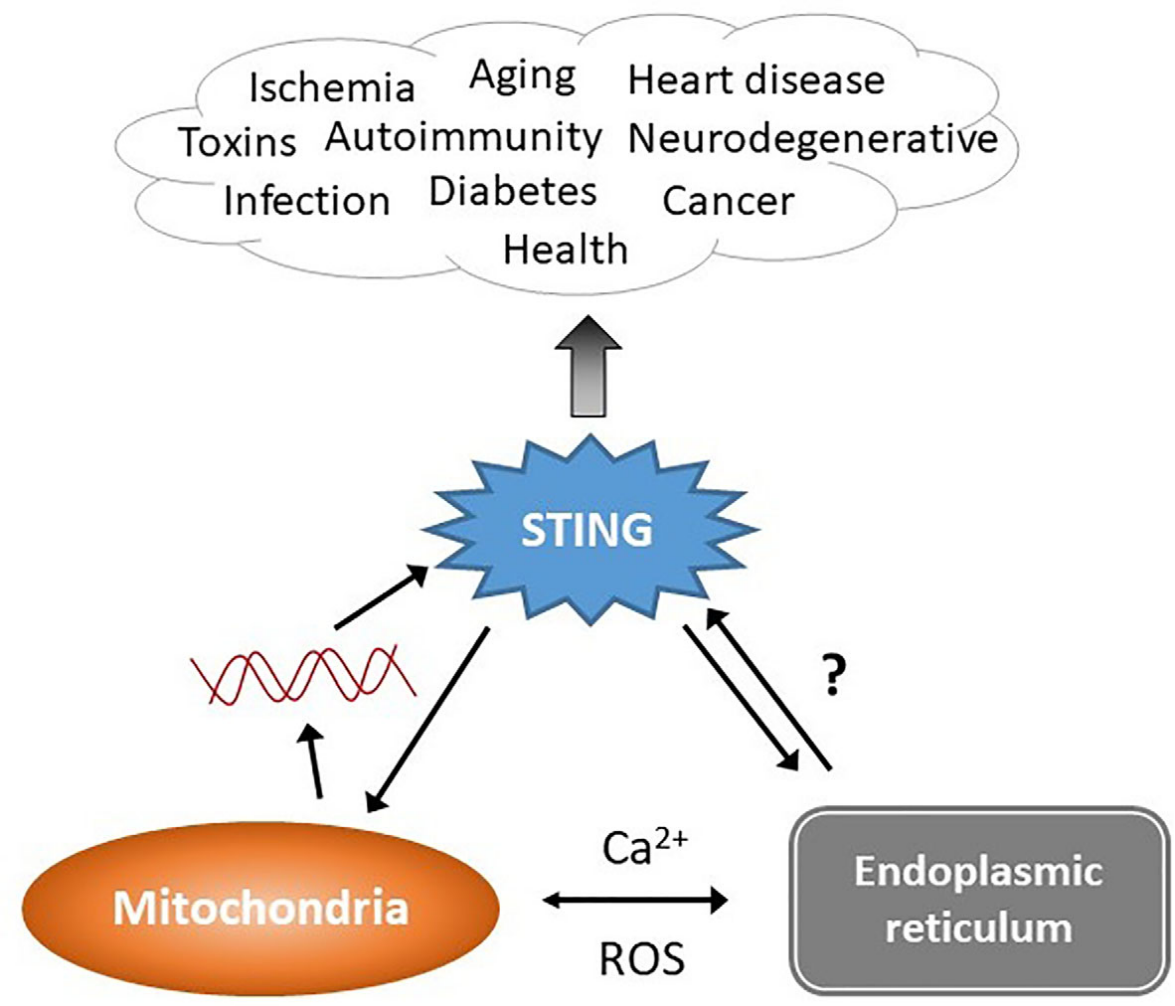
STING stimulation by stressed organelles: an interactive triad. STING plays a critical role in preserving health but also mediates disease, even in the absence of infectious triggers. Mitochondrial DNA (red lines) has recently emerged as a trigger of STING activation. The endogenous ligand mediating the ER-STING reciprocal relationship is not clear. The endoplasmic reticulum (ER) and mitochondria share a very close anatomic and functional relationship, and together modulate homeostatic and pathologic levels of intracellular calcium (Ca^2+^) and reactive oxygen species (ROS). This relationship may generate the “missing ligand” for ER stress-mediated STING activation *via* mitochondrial DNA release.

Nature has a dramatic capacity for repurposing. The same pattern recognition receptors (PRRs) that recognize pathogen-associated molecular patterns (PAMPs) on invaders such as bacteria and viruses also respond to endogenous products, particularly those generated during tissue damage (damage associated molecular patterns or DAMPs (1)). For example, Toll Like Receptor 4 (TLR4) not only recognizes bacterial cell wall lipopolysaccharide, but also responds to components of the extracellular matrix such as fibrinogen and fibronectin that are released during infectious and immune damage (2, 3). Not all endogenous PRR stimuli are from infection-mediated tissue damage, as PRRs are also involved in normal physiologic function. For instance, TLRs direct development and cell fate in *Drosophila*, *C. elegans* and mice (4). In the brain, TLRs modulate neuronal connectivity and function (5). “Sterile” PRR engagement also drives pathology: In autoimmune disease (e.g. lupus), nucleotide-activated receptors such as TLR7 (RNA) and TLR9 (DNA) respond to material released from apoptotic cells, regulating inflammation in a cell-specific manner (6, 7). Most PRRs, such as TLRs, C-lectin type receptors, Retinoic acid-inducible gene I (RIG-I) like receptors, inflammasomes and Nod-like Receptors (NLRs), reside on the plasma membrane, within endosomes or within the cytosol. These locations prime PRRs to respond to both pathogen and endogenous products in the extracellular space or cytosol. In contrast, Stimulator of Interferon Gene (STING) resides in the endoplasmic reticulum (ER), particularly in ER-mitochondrial appositions, with its triggering face to the cytosol (8). This unique location is not only useful for detecting cytosolic invaders; the organelle associations position STING to respond to alarm signals generated by the mitochondria and ER. Interestingly, the multi-molecular inflammation generating machinery triggered by RIG-I family PRRs and inflammasomes, including the lynchpin mitochondrial anti-viral signaling protein (MAVS) aggregates at the mitochondria (9); The MAVS C-terminal transmembrane domain inserts in the outer mitochondrial membrane where it nucleates the formation of filamentous signaling platforms (10, 11). Involvement of, and crosstalk between these organelles may critically contribute to PRR signaling by increasing signal amplitude and providing further context (intracellular stress). In this review, we will focus on the crosstalk between STING, ER and mitochondria. Although many previous lines of inquiry have focused on dyads in this triangle (**Figure 1**, conceptual framework for this review), we posit that DAMP-stimulated STING signaling may reflect three-way communication between these organelles and STING.

## The Key Players: cGAS and STING

Viruses depend exclusively upon host building blocks and machinery to produce the RNA and DNA strands that encode their genomes. During some portion of their lifecycle (e.g. uncoating, creating progeny), viral genomic nucleic acid will be present in the host cytosol. Thus, sensors of cytosolic nucleic acids such as STING constitute a vital defense that has been in place across 600 million years of evolution (12, 13). STING directly binds cyclic-di-nucleotides (CDNs). STING also “senses” cytosolic dsDNA indirectly *via* its “partner” in detection, cyclic-GMP-AMP (cGAMP) synthase (cGAS); upon binding dsDNA, cGAS generates endogenous cyclic-di-nucleotides that serve as the actual STING ligands. Although multiple molecules may detect cytosolic dsDNA in addition to cGAS (e.g. Gamma interferon inducible protein 16 (IFI16), Dead box helicase 41 (DDX41)), cGAS is the primary dsDNA-sensor required for STING activation by dsDNA (14, 15). The role of these other sensors remains unclear, though IFI16 promotes cGAS activation and enhances STING phosphorylation, translocation, and Tank binding kinase 1 (TBK1) recruitment (16, 17). DDX41 may promote IFN-induced cGAS expression (18). cGAS senses cytosolic DNA, but in the resting state in macrophages and other cell types, the vast majority of cGAS resides inside the nucleus, sequestered by chromatin (19–21). One study from Barnett et al. also placed cGAS at the plasma membrane *via* an N-terminal phosphoinositide interaction; the basis for this discrepancy with the other studies is not clear (22). Recent cryo-electron microscopy structural data has elucidated the inhibitory relationship between chromatin and cGAS that prevents self-recognition: an acidic patch on the nucleosome histone H2A-H2B heterodimer occupies the dsDNA binding site, preventing cGAS activation and dimerization (23–27). This new information begs the question of how nuclear cGAS responds to pathogenic challenges, as described in the setting of HIV2 recognition (28). Perhaps nuclear proteins such as non-POU domain-containing octamer-binding protein (NONO) extract cGAS from the nucleosomes during infection to enable response to nucleus-located viruses. The mechanism by which nuclear cGAS access cytosolic dsDNA is equally mysterious. Clarification of this process awaits further study.

cGAS recognizes dsDNA at least 45nt in length, retrovirus-transcribed cDNA and Y-form DNA with an overhanging stretch of guanines (29–31). Retroviral triggers of cGAS include both pathogen-derived nucleic acid and potentially endogenous retroviruses (30, 32). cGAS directly binds the DNA deoxyribose sugar phosphate backbone, explaining the sequence-independence of recognition (33). DsDNA recognition is both length and concentration dependent, requiring a size ~1 kb at more physiologic levels (31). DsDNA-bound cGAS forms liquid-phase like droplets, potentially increasing local DNA concentration and valency (34). The formation of two by two structures (2 strands of DNA, 2 cGAS molecules) induces a conformational change in cGAS, which activates its nucleotidyl-transferase enzymatic activity (35). Using ATP and GTP as initial substrates, cGAS catalyzes the production of an asymmetric cyclic-di-nucleotide product with a 2’-5’ phosphodiester bond between the 2’ hydroxyl of GMP and 5’ phosphate of AMP, and a 3’-5’ phosphodiester bond linking the 3- hydroxyl of AMP back to the 5’ phosphate of GMP, referred to as 2’3’-cGAMP (36, 37). STING also binds the bacterial second messenger cyclic-di-GMP, which was the first identified ligand for STING, cyclic-di-AMP produced by Gram-positive bacteria such as *Staphylococcus* and *Listeria*, and bacterial origin 3’3’-cGAMP (38–40). Interestingly, the affinity of STING for bacterial products is much lower (>1–2 logs) than the endogenously generated 2’3’-cGAMP, suggesting an anti-viral evolutionary priority (37, 41, 42). Another possibility is that bacteria serendipitously coopted STING’s CDN-binding capacity to enhance type I interferon (IFN) production by the host cells, which benefits multiple bacterial species (13, 43, 44).

**Figure 2:**
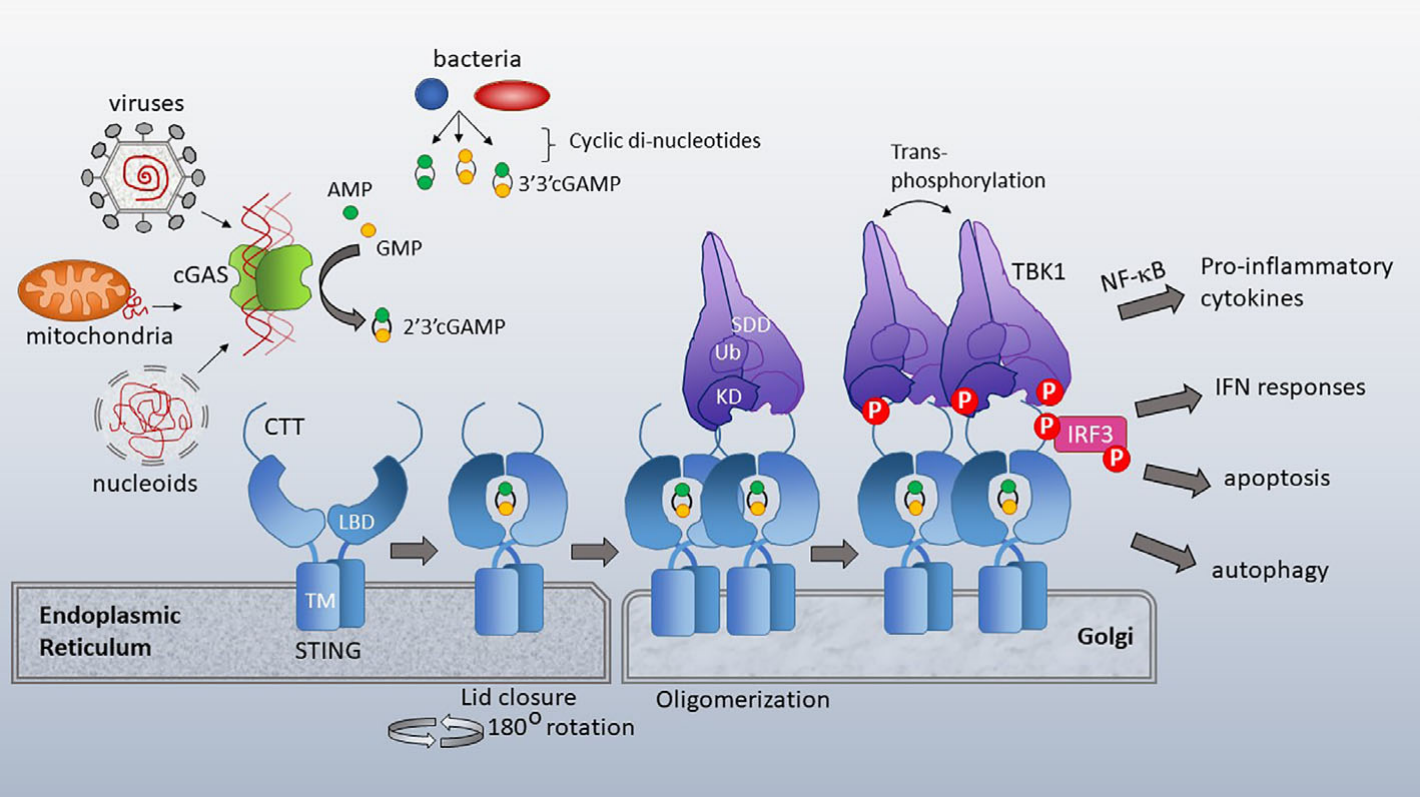
cGAS and STING activation. Cytosolic dsDNA from viruses, mitochondria or nucleoids formed during nuclear breakdown bind cGAS, triggering its catalytic formation of 2’3’ cGAMP. 2’3’ cGAMP serves as a ligand for STING, which resides in the ER with its ligand binding domain (LBD) facing the cytosol. TM=transmembrane domain. Bacterial cyclic-di-nucleotides, such as cyclic-di-AMP, cyclic-di-GMP and 3’3’ cGAMP also bind STING. Upon ligand binding, the cytosolic domains of STING close over the di-nucleotide ligand and rotate 180 degrees, enabling lateral stacking. STING translocates to the Golgi where it oligomerizes. This oligomerization enhances trans-phosphorylation of the STING CTT (C terminal tail)-associated TBK1 family kinases. TBK1 has a scaffold and dimerization domain (SDD), ubiquitin like domain (Ub) and Kinase domain (KD). Activated TBK1 phosphorylates the STING CTT, enabling recruitment and subsequent phosphorylation of IRF3. TBK1 family kinases also activate signaling pathways leading to NF-κB nuclear translocation. STING activation has diverse immune stimulatory outputs including pro-inflammatory cytokine responses (via NF-κB), interferon responses (via IRF3), apoptosis and autophagy. STING/TBK1 structural cartoon adapted from (51).

In its inactive state, the STING molecule, which has 4 membrane-spanning helices, resides in the ER plasma membrane as a dimer, with its v-like CDN binding domain facing the cytosol (45). Upon binding cGAMP, STING undergoes a conformational change that enables a lid-like 4-pass beta sheet to flop down over the CDN binding site in a “closed” position, and rotates the cytosolic portion 180 degrees. This rotation allows for higher order STING oligomerization and lateral stacking (46–48). CDNs such as cyclic-di-GMP stabilize STING in a more open position vs. cGAMP, perhaps explaining their lower affinity and activity (41, 49). In most vertebrates, with few exceptions, this lid also contains a flexible extended random coil C-terminal tail (CTT, amino acids 341–379 in humans) that has binding sites for TBK1 family kinases (TBK1 and Inhibitor of nuclear factor kappa B kinase (IKK)ε) (50). Some TBK1 associates constitutively with STING dimers, but the TBK1 dimer’s kinase domains face away from each other, preventing cis-phosphorylation (51). The higher order structures promote TBK1 trans-phosphorylation and activation. STING phosphorylation on Thr376 enhances TBK1 association ~20-fold (47). TBK1 also phosphorylates STING on Ser365/366 (mouse/human), forming a binding site for the interferon-regulatory transcription factor IRF3 (52). TBK1 phosphorylates IRF3, enabling the dimerization of IRF3 required for nuclear entry (53). The kinase domain of STING-attached TBK1 cannot access the cis-IRF3 molecule and thus relies on the close proximity of other TBK1 molecules to accomplish IRF3 activation (51). Changes in STING localization appear to be very important for specific activation steps in the TBK1-IRF3 signaling pathway. Following CDN binding, STING transits *via* the ER Golgi intermediate compartment (ERGIC) to the Golgi in a Coat protein complex II (COPII), Sar1 GTPase, ADP ribosylation factor (Arf1)-dependent manner (54, 55). Blocking this transition with agents like Brefeldin A, or the *Shigella flexneri* IpaJ protein prevents TBK1 association and phosphorylation (55, 56). Activated TBK1-STING then clusters together in peri-nuclear punctae where IRF3 is phosphorylated in multi-molecular “signalosome” complexes. These multi-molecular complexes also result in the activation of nuclear factor kappa-B (NF-κB), which then cooperates with IRF3 to induce the prototypic IFN gene *IFNB1* and promotes pro-inflammatory cytokine transcription. For a summary of cGAS-STING activation, see **Figure 2**.

STING is most widely known for IFN stimulation, and secondarily pro-inflammatory cytokine stimulation *via* NF-κB. However, STING triggers multiple signaling cascades: STING activates MAP kinase signaling, STAT6, inflammasomes (e.g. NLR family pyrin domain containing 3 (NLRP3)), autophagy and apoptosis (57–62). STING also suppresses translation, inhibiting viral infection, independently of eukaryotic initiation factor 2α (63). The detailed mechanisms by which STING initiates these different functions remain to be elucidated. Consider NF-κB activation for example: multiple reports document the necessity of the CTT and TBK1 for NF-κB activation (50, 58, 64). TBK1 does appear to be critical for IRF3 activation, an observation that has borne up over time. However, in myeloid cells, either TBK1 or IKKε can mediate NF-κB activation (50). TBK1 or IKKε activates Mitogen-activated protein kinase kinase kinase 7 (TAK1) and thus inhibitor of nuclear factor kappa-B kinase subunit (IKKβ/IKKα), resulting in inhibitor of κB (IκB) phosphorylation, IκB proteasomal degradation and nuclear factor kappa B (NF-κB) nuclear translocation (58). In myeloid cells, STING-dependent NF-kB activation did not require Tumor necrosis factor associated factor 6 (TRAF6). An alternatively spliced form of STING lacking the CTT, designated as MITA-related protein (MRP), functions as a dominant negative of IFN production yet activates NF-κB signaling independently of TBK1 (65). In the setting of genotoxic DNA damage, STING activates NF-κB independently of cGAS (and cGAMP) *via* association with p53, TRAF6, and IFIT16 (66). The zebrafish STING CTT contains an extra tail-end module that enhances NF-κB activation through increased recruitment of TRAF6 (67). In zebrafish, TRAF6 was essential for both NF-κB and IRF3 activity. Interestingly, TBK1 deletion in zebrafish only decreased IFN production by ~60% and NF-κB not at all, suggesting some flexibility and substitution capacity in STING modular functional domains. Together, these studies support context-dependent requirements for TBK1-family kinases and specific NF-κB activating pathways.

Evolution poses other questions regarding primordial STING function and NF-κB activation. Recognizable STING and cGAS orthologs are present in unicellular choanoflagellates, pre-dating NF-κB (68). *Nematostella vectensis*, a sea anemone that diverged from human ancestors >500 million years ago, possesses a STING molecule with only ~29% aa identity to human STING, but virtually an identical crystal structure to the STING core (12). Interestingly, the Mab-21 domain containing nucleotidyl-transferases such as cGAS date back as far as STING, but the *Nematostella* homologue makes 3’3’-cGAMP, not 3’2’-cGAMP (12). *Nematostella* cGAS also lacks the zinc-binding region present in vertebrates that is required for dsDNA binding. TBK1 and NF-κB also date back to *Nematostella*, but the CTT only developed in vertebrates, so it is not clear if primordial (pre-CTT) STING stimulates NF-κB in a TBK1-dependent manner (13). Thus, even though all the components were present early in evolution, their interactions and scope of activity remain a mystery.

The mechanisms by which STING induces autophagy are also not entirely clear. It has been proposed that the STING core moiety (lacking the CTT) contains a primordial autophagy function: In reconstitution experiments in HEK293 cells, the STING core was sufficient for initiating autophagy upon stimulation with exogenous cGAMP. Further, this core autophagy function exerted anti-viral activity – particularly against DNA virus such as Herpes Simplex Virus 1 (HSV1), but not the RNA virus Sendai virus (SeV). In this report, upon transit to the Golgi, the CTT-deleted STING core initiated Microtubule-associated protein 1A/1B-light chain 3 (LC3) lipidation through a non-canonical mechanism involving WD repeat domain phosphoinositide-interacting protein 2 (WIPI2) and Autophagy related 5 (Atg5), but not Unc-51 Like Autophagy Activating Kinase 1 (ULK1) and the Vacuolar protein sorting 34 (VPS34) complex. Although Mammalian target of rapamycin (mTOR) regulates autophagy, STING did not dephosphorylate or inhibit mTOR (54). However, in a recent report examining S365A and CTT deletion mutants in mice, HSV-1 viral resistance was IFN independent, but required the CTT and TBK1 for both autophagy and viral resistance. S365 phosphorylation was also important for enhancing NF-κB activation in macrophages (64). Multiple components of the cGAS-STING activation cascade interact with autophagy related molecules and pathways: TBK1 has been noted to activate mitophagy *via* phosphorylation of Calcium Binding And Coiled-Coil Domain 2 (NDP52), p62, TAX1BP1 and optineurin (69). Following translocation to the Golgi, STING co-localizes with p62, LC3 and Atg9a (59). cGAS may also participate in autophagy induction independently of STING: cGAS binds Beclin1, releasing Run domain Beclin-1 interacting and cysteine-rich containing (RUBICON, a potent negative regulator of autophagy) thus stimulating autophagy (70). In summary, multiple signaling routes link STING-cGAS signaling with autophagy, and activation of any particular pathway(s), or dependence upon specific STING moieties, may be context-dependent.

STING also stimulates apoptosis and cell death *via* multiple mechanisms (71). During “intrinsic” apoptosis, mitochondria form Bcl2 Associated X (BAX)/Bcl2 antagonist killer 1 (BAK)-dependent micropores, resulting in mitochondrial outer membrane permeabilization, release of cytochrome c, and caspase activation (72). BAX/BAK macropores subsequently enable herniation of inner mitochondrial membranes and extrusion of mitochondrial DNA (mtDNA) (73). STING promotes phosphorylation of receptor-interacting protein kinase 3 (RIP3), which activates p53 upregulated modulator of apoptosis (PUMA, another pro-apoptotic Bcl2 family member), leading to mitochondrial outer membrane permeabilization (74). IRF3 and p53 also coordinately upregulate PUMA and Noxa (60). Moreover, activated IRF3 binds BAX directly *via* its BH3 domain, stimulating apoptosis (75). STING activation also leads to mitochondria-induced apoptosis indirectly *via* ER stress and its multiple pro-apoptotic programs (more on this connection below and in (71)). When apoptosis is inhibited by infection or genetically, STING-dependent type I IFN and TNF promote regulated necrosis or “necroptosis” (76, 77). Depending upon STING signaling strength and cell type, STING trafficking to lysosomes following autophagy induction results in lysosomal permeabilization and so called “lysosomal cell death” (61). Lysosomal rupture induces potassium efflux, and secondary NLRP3 activation, stimulating pyroptosis (61, 78). It should be noted however, that STING-inflammasome cooperation does not invariably increase pyroptosis (62).

Both cGAS and STING are subject to multiple types of transcriptional and post-translational modifications and regulation, reviewed extensively elsewhere (79–81), with a few examples presented here. IFN increases cGAS and STING expression, driving a positive feedback loop (18, 82). Both cGAS and STING expression are suppressed by DNA methylation in many tumors (83). Palmitoylation of STING in the Golgi is essential for its oligomerization and activity (84). cGAS can be inhibited by Protein Kinase B (Akt) phosphorylation and glutamylation (TTLL4 and TTLL6) (85, 86). Complex ubiquitination can activate or inhibit cGAS and STING by targeting them for degradation (79). The autophagic flux stimulated by STING may facilitate its lysosomal destruction post-stimulation in an ULK1-dependent manner (87). The same pro-apoptotic caspases stimulated by cGAS-STING operate to inhibit their activation: During intrinsic apoptosis, initiator and effector caspases (Caspase 9 and Caspase 3) result in cleavage of cGAS, STING and IRF3, thus limiting further STING signaling (88, 89). Inflammasome processed caspase1 also cleaves cGAS and inhibits its enzymatic activity following Gasdermin-D dependent K+ influx (90, 91). Regulation of STING/cGAS activity by ROS and calcium will be described below.

## STING in the “Sterile” Pathology of Disease States

**Figure 3:**
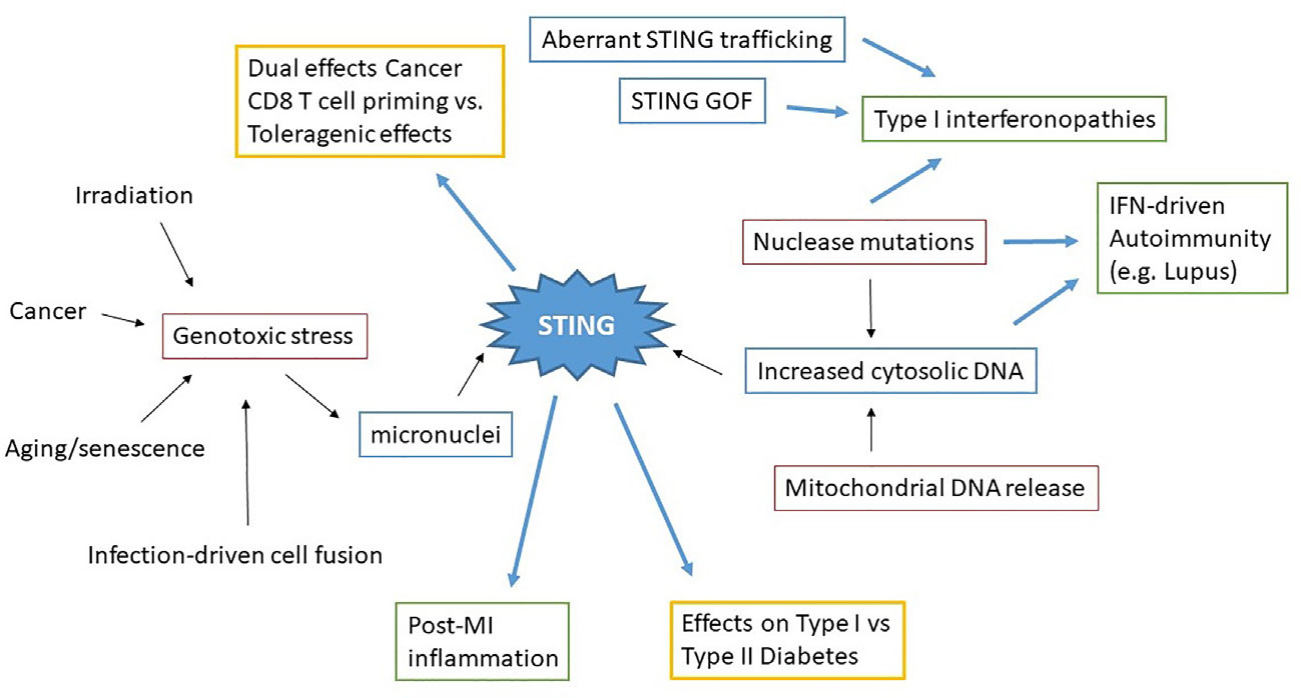
Concept map of STING in sterile pathology. Irradiation, cancer, aging/senescence and infection can drive genotoxic stress, resulting in the generation of micronuclei. Nuclease mutations, deficiencies, and mitochondrial DNA release lead to increased cytosolic dsDNA. These immediate drivers of cytosolic dsDNA are in red boxes. STING aberrantly activated through these processes, as well as STING mutations and altered STING regulation (blue boxes) all result in pathologic disease states. Excess STING-dependent IFN and inflammatory cytokines contribute to pathology (green boxes) in Type I interferonopathies, autoimmunity and post-MI (Myocardial Infarction). However the effects of STING on other types of pathologies (Cancer, Diabetes) can vary (yellow boxes) depending upon the specific situation.

Although cGAS and STING are poised to respond to pathogens, increasing evidence supports their critical role in a number of “sterile” physiologic and pathologic conditions, including cancer, heart disease, diabetes, neurodegenerative disease, lupus as well as normal aging/cellular senescence (**Figure 3**) (92–98). For example, gain of function mutations in STING and diminished nuclease activity lead to distinctive IFN-driven autoinflammatory conditions such as STING Associated Vasculopathy presenting in Infancy (SAVI) and Aicardi Goutieres syndrome, respectively (93). Increased STING trafficking to the Golgi (and sustained activation) results in autoimmunity in COPA syndrome (99). Lupus patients show a strong type I IFN signature in their peripheral blood, and a sub-group of lupus patients (~15%) has elevated circulating cGAMP (100, 101). However some autoimmune conditions dependent upon other PRRs may be regulated by STING and worsen with STING deficiency (102, 103). In regards to cancer, STING essentially mediates the anti-tumor effect of radiation (104). IFN promotes maturation and antigen presentation by CD8α+ dendritic cells (DC) and thus priming and activation of tumor infiltrating CD8 T cells and Natural Killer (NK) cells (49, 105). Related to these consequences of STING activation, STING agonists have shown promise as anti-cancer therapeutic agents (106). On the other hand, STING can promote toleragenic responses *via* Indoleamine-pyrrole 2,3-dioxygenase (IDO), especially with low antigenicity tumors, and induce the T cell exhaustion stimulus Programmed death ligand 1 (PDL1) (49, 107, 108). Inter-tumor cell cGAMP transfer and subsequent STING activation can also facilitate metastasis (109). Regarding cardiovascular disease, cGAS critically mediates inflammation post-myocardial infarction (MI), and induces CXCL10, iNOS expression and M1 differentiation. In this setting, STING or cGAS deficiency improved post-MI survival (110). STING activity may attenuate type I diabetes, but exacerbate type II diabetes and the associated metabolic syndrome (97, 111, 112). The dual positive and negative effects of STING on health mandate caution in modulating STING activity therapeutically.

One of the major questions posed by these observations relates to the source of “endogenous” STING/cGAS ligands in sterile pathology. In health, self DNA might be expected to be sequestered in the nucleus and within mitochondria. However, it has become clear that nuclei and mitochondria are not as “air tight” as previously thought. To maintain the status quo in health, nucleases patrol the cytosol and extracellular milieu, degrading rogue cytosolic nucleic acids. Even in the face of this nucleotide cleanup crew, increasing evidence suggests mtDNA and under certain conditions, nuclear and extracellular DNA serve as stimuli for STING. Extracellular dsDNA from apoptotic cells can be taken up by endocytosis, or in the forms of microvesicles and exosomes (113, 114). Internalized dsDNA translocates from lysosomes (especially if deficient in DNAse) into the cytosol (115). Extracellular cGAMP that evades Ectonucleotide pyrophosphatase/phosphodiesterase family member 1 (ENPP1) degradation can also be taken up by and stimulate cells (116). Studies in senescence, infection, neurodegenerative diseases, cancer and lupus have greatly elucidated the generation (and recognition) of mitochondrial and genomic DNA ligands. Genomic DNA will be discussed briefly, but the remaining focus will be on mtDNA.

Without killing the involved cell, genotoxic stress can result in partial breakdown of the laminin nuclear envelope structure and extrusion of so-called “micronuclei” into the cytoplasm (117). In senescence, telomere dysfunction and breakdowns in DNA repair lead to genotoxic stress. In cancer, genotoxic stress may result from aberrant mitosis, mutation in DNA-repair enzymes as well as exogenously applied radiation. Micronuclei only form during cell division, and breakdown of the micronuclei envelope during mitosis is required for recognition (117, 118). This damaged genomic DNA in micronuclei then becomes an available substrate for cGAS (95). It is unclear how the extra-nuclear DNA avoids nucleosomal inhibition of cGAS, unless the DNA dissociates from histones. The chromatin state in micronuclei is not well described. Once cytosolic micronuclei dsDNA stimulates cGAS, the resulting cGAMP can also transfer across cellular boundaries *via* gap junctions (109, 119). For instance in cancer, cGAMP from tumor cells transfers to STING-expressing myeloid and B-cells that produce natural killer cell-stimulating IFN (120). One bacterium, *Burkholderia pseudomallei*, induces cell fusion, genomic instability and aberrant mitosis resulting in micronuclei formation. Interestingly, in this case, the micronuclei triggers STING activation and IFN transcription but not IFN protein, and STING/autophagy-dependent cell death. Polyethylene glycol induced cell fusion also triggers IFN gene expression and autophagy, suggesting a critical role for the cell fusion process (121). Micronuclei detected in human Huntington’s disease embryonic stem cell-derived neurons have been linked to inflammation and autophagy (122).

## Further Cues on Endogenous Ligands From Lupus and Related Conditions: Important Roles for Nucleases and Mitochondrial DNA

Discovery of the linkage between nuclease deficiencies and type I interferonopathies has thrown the requirement for nucleotide “clean-up” into sharp relief. The cytosolic nuclease TREX1/DNAseIII was one of the first described molecular associations with type I IFN-driven diseases (123, 124). TREX1 deficiency and mutations have been implicated in Aicardi-Goutieres syndrome (AGS), Familial chilblain lupus, systemic lupus erythematosus (SLE) and retinal vasculopathy with cerebral leukodystrophy (RVCL) (123, 125–127). Full deletions are more likely to be associated with the severe early-onset manifestations, as in AGS, whereas heterozygous deficiencies are more common in complex milder or later onset conditions, such as familial chilblain lupus (126). AGS also results from defects in RNAse H2, which cleaves RNA from DNA to decrease DNA damage, and SAM and HD Domain Containing Deoxynucleoside Triphosphate Triphosphohydrolase 1 (SAMHD1), a dNTPase that acts at stalled replication forks and regulates reverse transcription to cDNA (128–130). Mice deficient in extracellular DNAseI develop high titer anti-nuclear antibodies and glomerulonephritis (131). Lysosomal DNAseII deficiency is embryonic lethal in mice, but completely rescued by cGAS or STING absence. Interestingly *DNaseII*-/-*Ifnar* (type I IFN receptor)-/- mice survive to adulthood, but develop rheumatoid arthritis-like disease, most likely reflecting the NF-κB stimulating activity of STING (132). TREX1, which is located in the cytosol and localizes to the ER, is active against ssDNA, nicked end dsDNA, and retroviral cDNA (produced for instance during HIV-1 infection) (133). TREX1 may also degrade endogenously transcribed retroviral elements (124, 134). Mutations in the DNAse region are mostly associated with AGS, whereas DNAse intact C-terminal truncations have been identified in RVCL and SLE (93). The non-DNAse TREX1 functions may be mediated through its association with and regulation of oligosaccharyltransferase, as C-terminal TREX1 mutations result in production of large amounts of immunostimulatory free glycans (135).

The association of SLE-like type I IFN driven diseases with these rare nuclease mutations serves as proof of principle regarding the importance of endogenous DNA in driving autoimmune disease. Work over the last decade has shed light on pathogenic mechanisms that may be more prevalent. Cells from lupus patients, most prominently neutrophils tend to extrude their nuclear contents in stringy structures rich in histones and dsDNA known as neutrophil extracellular traps (NETs). These NETS also contain inflammatory proteins such as Cathelecidin LL37 antimicrobial peptides and High Mobility Group Box 1 (HMGB1) (136). Wang et al. reported the presence of mitochondrial (mt)DNA in ex-vivo stimulated NETs and increased anti-mtDNA antibodies in lupus patients. These antibodies correlated with IFN scores and disease activity scores, including lupus nephritis scores, better than the standard anti-dsDNA. This group also found mtDNA in NETs in SLE subject kidney biopsies. MtDNA-anti-mtDNA complexes were strong stimulators of plasmacytoid DC IFN, even more so than anti-dsDNA. This group then performed a proof of concept trial with metformin, which decreases mitochondrial respiratory chain complex I and NADPH oxidase activity, suggesting that oxidation played a key role in pathogenesis (137). MtDNA lacks protective histones and DNA repair enzymes present in the nucleus and thus, is more susceptible to oxidation. Oxidized DNA is also resistant to TREX1 degradation, making this a plausible scenario (138). This study concluded the IFN was TLR9-dependent, but the evidence was very indirect.

**Figure 4:**
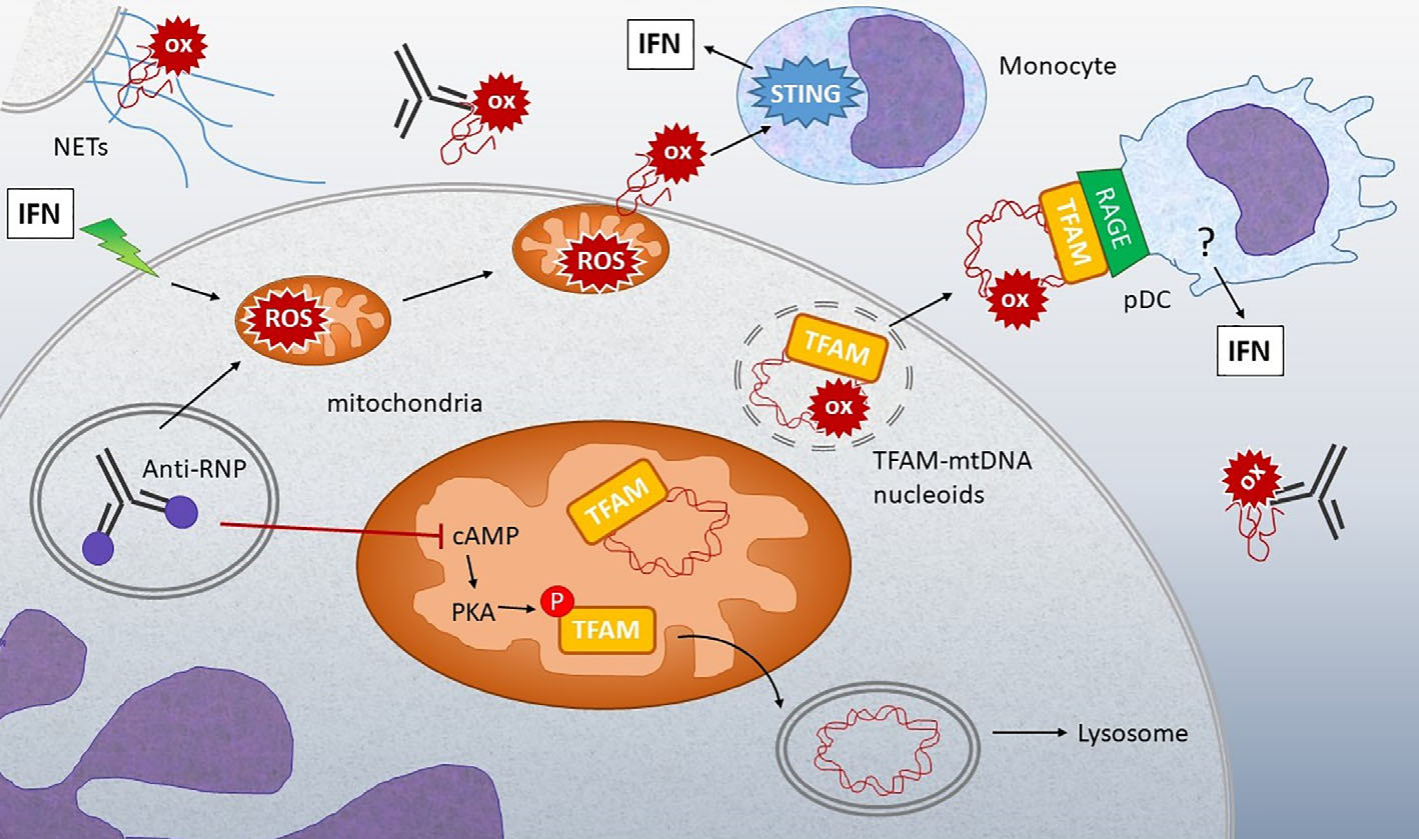
Connection between oxidized mtDNA and Lupus. Stimulation of IFN-treated neutrophils or neutrophils from lupus patients with anti-RNP immune complexes can lead to release of oxidized (ox) mtDNA by multiple mechanisms: 1) Stimulation of NETosis, with extrusion of DNA containing oxidized mtDNA. 2) Increased mitochondrial ROS leads to membrane translocation and extrusion of oxidized mtDNA into the extracellular milieu. 3) Anti-RNP and type I IFN decrease the levels of cyclic AMP (cAMP), a second messenger required for activation of protein kinase A (PKA), which normally phosphorylates TFAM, enabling its release from mtDNA. When TFAM is released, the mtDNA can then go to the lysosome for degradation. If PKA is inhibited, TFAM remains associated with mtDNA in nucleoids that accumulate in mitochondria and then are released from the neutrophils through unclear mechanisms. Extracellular oxidized mtDNA is sensed by monocytes in a STING-dependent manner and internalized by pDC *via* RAGE receptors. Downstream of RAGE, the IFN-generating sensor in pDC is unclear, although both oxidized and non-oxidized mtDNA stimulation of pDC is TLR9-dependent (139). The abundance of anti-mtDNA antibodies in lupus and correlation with disease support the critical involvement of these mechanisms in disease pathogenesis (137, 139, and 140).

Two further studies from 2016 further explored the relationship between oxidized mtDNA and lupus. Caielli et al. reported that neutrophils from healthy subjects that sustain mitochondrial damage extrude DNA. This damaged mtDNA dissociates from the mitochondrial transcription factor A mitochondrial (TFAM) molecule that packages it into nucleoids en route to lysosomes for degradation. Dissociation requires protein kinase A (PKA)-mediated TFAM phosphorylation. Exposure of either type I IFN-treated neutrophils or neutrophils from lupus patients to anti-RNP immune complexes decreased the cAMP required for PKA activation. The TFAM-associated nucleoids remained in the cytosol, became oxidized and were released from the cell through unclear means. TLR9 and RAGE participated in uptake of the TFAM-associated oxidized mtDNA nucleoids by DC, thereby stimulating IFN production. In support of this mechanism, oxidized mtDNA autoantibodies were present in a fraction of patients and oxidized mtDNA nucleoids visualized in SLE patient neutrophils (139). In the report by Lood et al, they tied oxidized mtDNA to STING-dependent IFN as follows: anti-RNP immune complex stimulation increased mitochondrial ROS. Mitochondrial ROS resulted in hypopolarization, translocation of mitochondria to the plasma membrane and release of oxidized mtDNA into the extracellular milieu. This oxidized mtDNA was a potent stimulus of IFN production by peripheral blood mononuclear cells (PBMC) and monocytic THP1 cells. In mice, injection of oxidized mtDNA induced IFN in a STING-dependent manner. Furthermore, lupus patient low-density granulocytes spontaneously released NETS enriched in mtDNA in a mitochondrial ROS-dependent manner. As proof of principle, they administered a mitochondrial ROS antagonist (mitoTEMPO) to mice continuously *via* a pump, decreasing disease severity in lupus prone MRL/lpr mice. MtDNA oxidation occurred independently of NADPH-oxidase, in *Nox* knockout mouse cells and chronic granulomatous disease patients (140). Together these reports firmly establish a link between mitochondrial ROS, oxidized mtDNA and IFN generation in lupus (summarized in **Figure 4**). Some of the differences, for instance TLR9 vs. STING dependence, might reflect species in some experiments (mouse vs. human) as well as mtDNA stimulated target cell (DC vs. PBMC and monocytes).

Beyond lupus, mtDNA appears to play a role in STING activation in other sterile diseases, such as cancer, and toxin-stimulated injury. Cisplatin-induced acute kidney injury depended upon mitochondrial damage and stimulation of STING. Interestingly, in this report, STING stimulated NF-κB but not type I IFN production, yet more evidence that different STING outputs can be uncoupled (141). In regards to cancer, oxidized mtDNA sensing by STING promoted the antitumor effect of irradiated immunogenic cancer cells (142). DC appropriated oxidized mtDNA released from dying irradiated tumors and then cross-presented antigen to cytotoxic CD8 T cells.

Mitochondria also mediate STING stimulation in a variety of infectious settings, in effect, functioning as both DAMP and PAMP. STING plays an unanticipated role in responding to RNA viruses, with mitochondria mediating the interaction. Interestingly, many of these viruses express mitochondria targeting proteins. Dengue virus M protein targets the mitochondrial membrane, forming pores that result in swelling and loss of membrane potential (143). NS2B3 cleaves mitofusins 1(Mfn1) and Mfn2, influencing the structure and function of mitochondria (144). Dengue-induced mitochondrial stress and damage results in release of mtDNA into the cytosol (145). NS2B3 also directly cleaves STING, limiting production of type I IFN (146). Encephalomyocarditis and influenza induce mtDNA release into the cytoplasm *via* viroporins, stimulating cGAS and DDX41-dependent immune responses (147). Although Herpesviruses are DNA viruses, the HSV1 gene product UL12, that depletes TFAM and results in enlarged mitochondrial nucleoids and mtDNA release, is essential for full IFN production and anti-viral activity (145). Different strains of *Mycobacterium tuberculosis* induce varying amounts of mitochondrial stress and mtDNA release stimulating cGAS/STING-dependent IFN (148, 149). *M. abscessus* induces IFN and NLRP3 activity *via* mitochondrial oxidative stress. In this setting, IFN and IL-1β exhibited a mutually inhibitory reciprocal relationship (150).

## STING and the ER: Cross Talk and Cross Regulation

**Figure 5:**
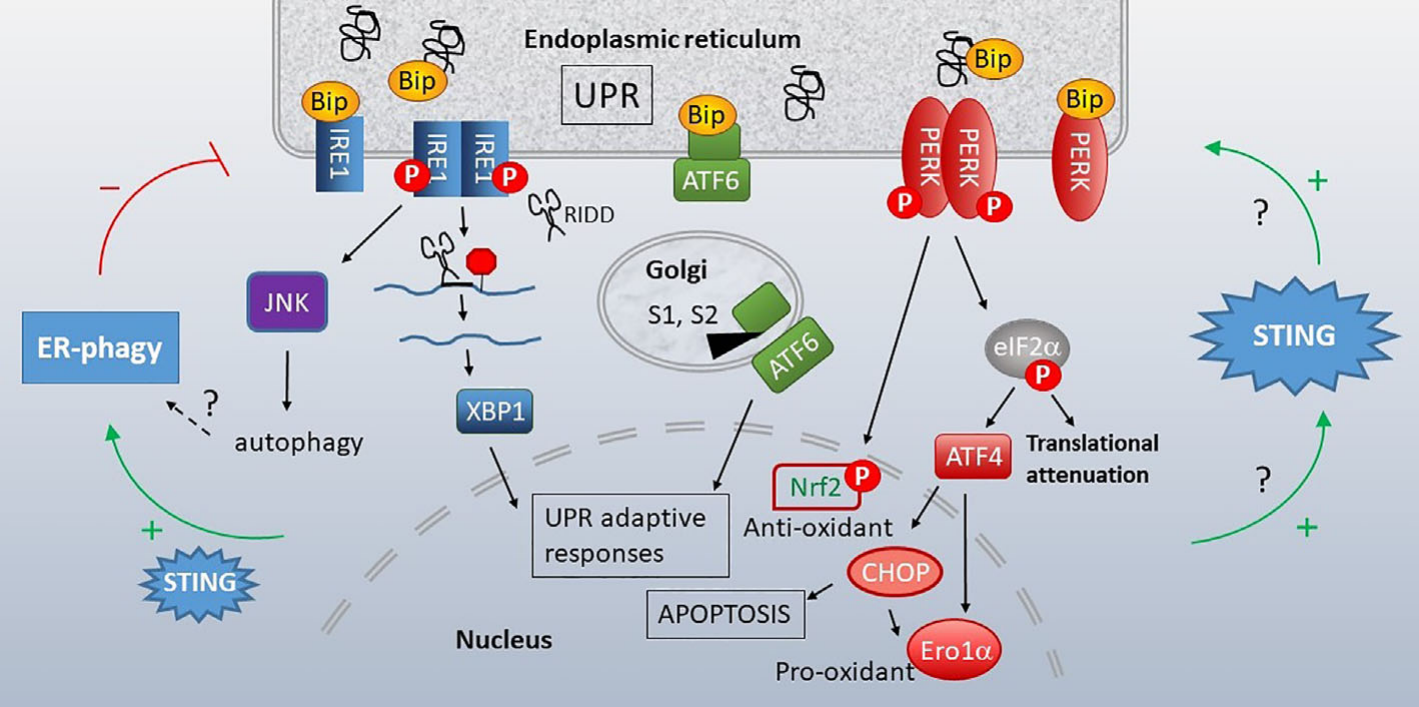
Unfolded Protein Response (UPR), STING and autophagy. When cellular insults or protein production demands compromise ER function, the ER initiates the UPR. Misfolding proteins bind the chaperone BiP, releasing it from three stress sensors, IRE1 (blue), ATF6 (green) and PERK (red). IRE1 is a bifunctional kinase/endonuclease that initiates JNK-dependent signaling and excises a 26bp stretch from the XBP1 mRNA, removing a premature stop codon *via* frameshift mutation. IRE1 also decreases ER load through more promiscuous endonuclease activity (RIDD). Upon release of BiP, ATF6 translocates to the Golgi, where S1 and S2 proteases generate an active transcription factor. PERK kinase phosphorylates eIF2α, resulting in global translational attenuation apart from select mRNAs such as ATF4. ATF4 promotes transcription of the pro-apoptotic transcription factor CHOP and Ero1α oxidoreductase. PERK also leads to nuclear factor erythroid 2–related factor 2 (Nrf2) nuclear translocation and resulting anti-oxidant responses. The UPR promotes STING activity and STING increases the UPR (right green arrows). This STING-dependent increase in UPR also enhances autophagy of ER components (“ER-phagy”, left side), which can limit ER stress responses. Many questions remain regarding the mechanistic details connecting STING, UPR and ERphagy.

The studies highlighted above describe a connection between mitochondria and STING activation *via* the release of mtDNA, which is oxidized in many cases. Increasing evidence also supports communication and cross-regulation between the ER and STING, in which an ER stress response known as the “Unfolded Protein Response” (UPR) takes center stage (UPR recently reviewed in (151) and in (152), **Figure 5**). The ER serves as the protein-producing factory of the cell. Different types of physiologic demands and insults that impact protein folding, including increased protein production, misfolding proteins, nutrient deprivation, hypoxia, calcium and oxidative dysregulation, all lead to induction of the UPR. The UPR encompasses three primary signaling arms set in motion by the activation of ER-membrane associated stress sensors, serine/threonine-protein kinase/endoribonuclease inositol-requiring enzyme 1 α (IRE1), Protein Kinase R-like endoplasmic reticulum kinase (PERK) and activating transcription factor 6 (ATF6). Very briefly, in the absence of stress, these sensors associate with an ER-protein folding chaperone GRP78 (Immunoglobulin heavy chain binding protein (BiP)). An overabundance of unfolded protein results in the release of these sensors from BiP, thus activating UPR signaling. IRE1 is both a kinase and endonuclease which processes the X-box binding protein 1 (XBP1) transcription factor mRNA, yielding the active transcription factor. XBP1 promotes production of ER chaperones, ER associated degradation (ERAD) proteins, and ER expansion. In certain settings, IRE1 also displays non-specific endonuclease function decreasing protein load, a process termed Regulated IRE1-dependent decay (RIDD). IRE1 kinase signals *via* NF-κB and JNK pathways, stimulating inflammation, autophagy and apoptosis. PERK is a kinase whose activity results in the phosphorylation of eukaryotic initiation factor 2 α (eIF2α). Phosphorylation inhibits global mRNA translation apart from select transcripts with 5’Cap-independent upstream open reading frames such as ATF4. PERK regulates amino acid acquisition, redox status and apoptosis *via* induction of the C/EBP Homologous Protein (CHOP) transcription factor. ATF6 is a proto-transcription factor that traffics to the Golgi following BiP dissociation. There, Site1 and Site2 proteases cleave ATF6 to an active transcription factor that induces ER chaperones and with XBP1, increases ER capacity. As the UPR accomplishes much of its adaptive program through gene transcription, it is often monitored experimentally by quantitating UPR target gene expression. Together these pathways re-establish proteostasis (proteome homeostasis) by decreasing protein load, at least temporarily, and enhancing ER function. If ER stress is profound or fails to resolve, these pathways trigger apoptosis.

The UPR intersects with, and activates pro-inflammatory signaling [extensively reviewed elsewhere (153–155)]. Moreover, cells undergoing ER stress respond to PRRs with synergistic cytokine production; thus, the UPR acts as an amplifier for pathogen recognition (153, 156, 157). In TLR4-stimulated macrophages, IFN-β was one of the most dramatically enhanced cytokines by ER stress (157). Further, using chemical UPR inducers and oxygen-glucose deprivation, we found that ER stress was sufficient for phosphorylation and nuclear translocation of IRF3 (158). Interestingly, IRF3 activation was only STING-dependent for certain types of ER stress induction, such as oxygen glucose deprivation and treatment with Thapsigargin, which induces ER stress *via* inhibition of the calcium-regulating sarco/endoplasmic reticulum Ca^2+^-ATPase (SERCA) pump (159). Oxygen glucose deprivation can also increase cytosolic calcium *via* modulation of SERCA, Ryanodine receptors (RyR) or other receptors (160, 161). Tunicamycin, an UPR-inducing N-linked glycosylation inhibitor resulted in IRF3 phosphorylation through a STING-independent, but ATF6-dependent mechanism (158). Calcium may be key for STING activation by the UPR, but increased cytosolic calcium, introduced with a calcium ionophore, was not sufficient in the absence of ER stress.

The linkage between ER stress and STING activation detected with *in vitro* pharmacologic manipulation was observed in several disease models: Patrasek et al. reported that in early alcoholic liver disease, alcohol induced ER stress, which resulted in STING activated IRF3 and IRF3-dependent apoptosis (162). In traumatic brain injury, a PERK inhibitor abrogated STING activation and ameliorated damage (163). In a report from Cui et al, *Mycobacterium bovis* STING activation of TBK1 and IRF3 was dependent upon ER stress (164). In this study, IRF3-BAX-initiated apoptosis required TBK1 activity. We found that during *Brucella abortus* infection, induction of the UPR was critical for STING activation and induction of the STING-dependent type I IFN program genes. However, this study also brought up an interesting, somewhat thorny observation: Induction of the UPR was STING dependent (lower in STING-/- cells), so there is reciprocal regulation. Further, type I IFN enhanced UPR induction (165). This type of reciprocal crosstalk was also described in a report on heart inflammation and fibrosis. Angiotensin II induced STING expression and increased IFN in cardiac myocytes in an ER stress dependent manner. In this study, STING-/- mice exhibited decreased ER stress following aortic banding (166). Moretti et al. described STING activated ER stress and autophagy induction in the setting of *Listeria innocua* infection. *Listeria* c-di-AMP stimulated STING, resulting in upregulation of ER stress markers, inhibition of mTOR and autophagy. In this particular type of autophagy, “ER phagy”, ER membranes that were autophagocytosed included ER markers, ER stress proteins and STING. They observed a yin-yang relationship between ER stress and autophagy: ERphagy reduced ER stress (especially the PERK pathway). Inhibition of autophagy (and increased ER stress) resulted in apoptosis. PERK deletion decreased IFN production and autophagy, suggesting an ER-stress feed forward mechanism (167). Putting these studies together, ER stress can induce STING activity and STING increases ER stress and ER stress-dependent autophagy.

A report by Wu et al. examined the molecular basis for the UPR-STING connection by focusing on a STING gain of function (GOF) mutant associated with SAVI (168). Patients afflicted with SAVI develop early onset vasculitis, rash and interstitial lung disease (169). SAVI is largely IRF3-independent in mice, suggesting non-IFN STING activities are important drivers of the inflammatory disease (170). Wu et al. found increased expression of UPR markers, BiP and CHOP in GOF human T cells, less so in B cells and not in macrophages, MEFs or fibroblasts (UPR was cell type-dependent). In the Jurkat cell line, the STING GOF mutant was not sufficient for UPR induction but synergized with T cell receptor (TCR) stimulated ER stress. In wild type murine T cells, TCR engagement typically induces ER stress, but not apoptosis. In the wild type T cells, the strong STING agonist DMXAA and TCR stimulation, but not DMXAA alone, significantly increased ER stress. Similarly, the GOF STING mutant synergized with TCR signaling to increase ER stress and IRF3-independent apoptosis. Furthermore, Wu et al. defined a requisite “UPR motif” within STING, in aa322–343, a highly conserved sequence encoding a helix on the exterior of the STING dimer, next to the CDN ligand-binding domain. Residues R331 and R334 were particularly important for UPR function. A deletion around the IRF3 binding site (343–354) abolished IFN, but not UPR or NF-κB outcomes (168).

## STING and Calcium

In addition to the UPR, a second related aspect of ER function, calcium regulation, has been implicated in STING activation and function. The ER serves as the primary calcium storage within the cell, maintaining a huge gradient across the ER membrane. ER calcium concentrations are estimated at 2mM with free calcium at 500μM, whereas cytoplasmic calcium is in the 100–100 nM range (171, 172). These high concentrations of calcium are critical for optimum function of the ER protein-folding machinery. ER-resident calcium binding proteins with low affinity but high capacity include the chaperones calreticulin, calnexin, BiP, grp94 and Protein disulfide isomerase (PDI) (173). Calreticulin and calnexin work with Erp57, the thiol disulfide isomerase, to form disulfide bonds and promote protein folding (174). Calreticulin and calnexin also direct protein trafficking through the ER and ERAD (175). The BiP ATPase prevents protein aggregation (176). Calsequestrins and Chromogranins further buffer ER calcium. Three families of proteins, SERCA, Inositol triphosphate receptors (IP3R) and RyRs, mediate the tremendous ER-cytosol gradient and regulate calcium release. Expression and relative roles of these proteins is cell-dependent. For instance, there are 3 SERCA genes with multiple splice variants, but SERCA2b is most widely expressed, has the highest calcium affinity of the SERCAs and is primarily responsible for maintaining high ER calcium (177). Type 1 IP3R is located throughout the ER but type 3 IP3R localize to the mitochondrial associated membranes (MAMs) and primarily transmit calcium to mitochondria (178). RyRs are expressed most prominently in muscle, but even at lower concentrations, they may exert strong effects, as RyRs release ~20x more calcium into the cytosol than IP3Rs (179). Stromal interaction protein 1 (STIM1) is a transmembrane calcium sensor that senses ER calcium levels through its EF hand and other calcium binding sites (180). When ER calcium is depleted, STIM1 translocates to the plasma membrane where it binds the Calcium release-activated calcium channel protein 1 (Orai1) resulting in capacitative calcium entry, also known as Store operated calcium channel (SOC) entry (181).

STING monomers share 2 Ca2+ binding sites when they form dimers, and a certain amount of cytosolic calcium appears necessary for activation (182). For instance, during dsDNA-stimulated STING activation in macrophages, calcium chelators such as BAPTA and mitochondrial calcium export inhibitors (CGP37157 sodium pump inhibitor) both reduce IRF3 and NF-κB activation (183). W-7, a potent calmodulin inhibitor also reduced STING activation by a pharmacologic STING-stimulating adjuvant (184). Early sensing of HCMV (human cytomegalovirus) and Sendai virus membrane perturbations and ensuing STING activation is calcium dependent (185). Cyclosporin A (the calcineurin inhibitor) decreases mitochondrial calcium release and STING-dependent IFN in macrophages (183). Short-term elevations in cytosolic calcium increase STING activity through the following mechanism: calcium binds and activates Calcium/calmodulin-dependent protein kinase II (CAMKII), which then phosphorylates 5’ AMP-activated protein kinase (AMPK) (186). AMPK represses ULK1, which phosphorylates (and negatively regulates) STING (87). However, saturating levels of cytosolic Ca^2+^ (as following ionomycin treatment) can also inhibit STING activation, so a happy medium is required by STING for optimal function (187). The importance of the calcium-STING connection has borne out in lupus: dipyridamole (a Ca channel blocker and cGMP phosphodiesterase inhibitor) reduces cytokine production in SLE T cells (188). CAMKIV is overexpressed in lupus nephritis and CAMK deficiency or inhibitors (e.g., KN-93) decrease disease in murine lupus models (189, 190).

Calcium homeostasis is also important for controlling STING location in its basal state and during activation. The ER calcium sensor, STIM1 physically interacts with and inhibits STING activation and translocation to Golgi (191). Exogenously increased STIM1 greatly decreases STING activation and UPR induction. The STIM1-STING inhibition is mutual, in that STING inhibits STIM1 translocation. When STING is absent, STIM1 enriches at the plasma membrane, and mediates increased calcium entry *via* SOC.

**Figure 6:**
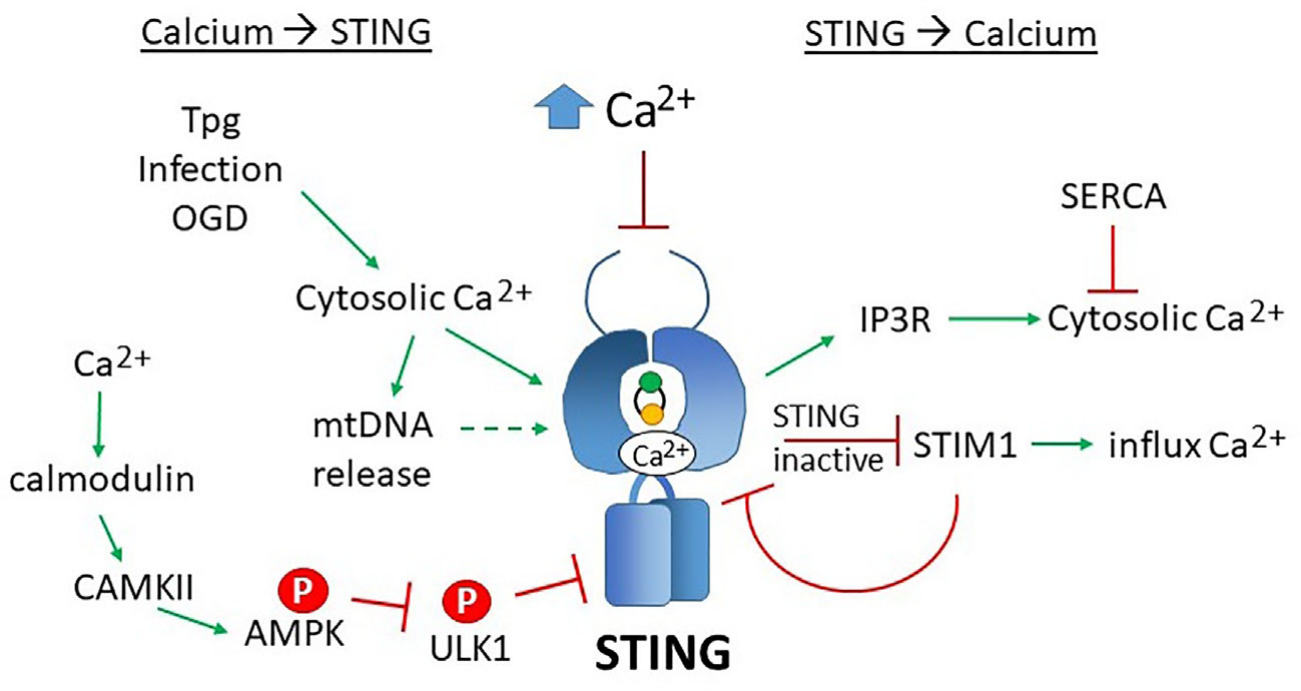
Reciprocal effects of calcium on STING activity and STING on calcium homeostasis. Increases in cytosolic calcium (Ca^2+^) enhances STING activity through multiple mechanisms: 1) calcium directly binds STING dimers, promoting cyclic-di-nucleotide signaling, 2) increased cytosolic calcium enhances mitochondrial DNA extrusion (thus triggering cGAS), and 3) calcium stimulated calmodulin activates CAMKII, which phosphorylated AMPK, which then inhibits ULK1, a STING inhibitor. SERCA pump inhibitors Thapsigargin (Tpg), infection and oxygen glucose deprivation (OGD) increase cytosolic calcium, thereby stimulating STING. The mechanisms underlying these observations are not yet established. Too much calcium (as in ionomycin treatment) inhibits STING activity. On the right, STING stimulates IP3R-dependent calcium release, a process that may be counteracted by SERCA activity. In its inactive state, STING sequesters STIM1 in the ER, preventing extracellular calcium entry. STIM1 reciprocally “tethers” STING to the ER, inhibiting its activity.

Not only does calcium regulate STING activity and location, but STING, in a reciprocal fashion, may regulate calcium levels. STING associates closely with ER SERCA pumps and mitochondrial calcium transporters VDAC1 and VDAC3 in the MAMs (192). Direct association between STING and IP3Rs increases cytosolic calcium release and drives lethal coagulation during sepsis (193). The STING GOF mutant (chronic STING activity) exhibits decreased ER Ca^2+^ release and lower influx across the plasma membrane. However, acute T cell receptor signaling and activation of the GOF mutant resulted in increased calcium-dependent ER stress. Exacerbating this effect with the SERCA pump inhibitor Thapsigargin (but not Tunicamycin) synergized with the STING GOF mutation in inducing apoptosis (168). Thus, STING may regulate calcium homeostasis and set thresholds for calcium-mediated signaling and apoptosis. For a summary of STING and calcium reciprocal regulation, see **Figure 6**.

## The ER Mitochondrial Connection: Communication *via* ROS and Calcium

**Figure 7:**
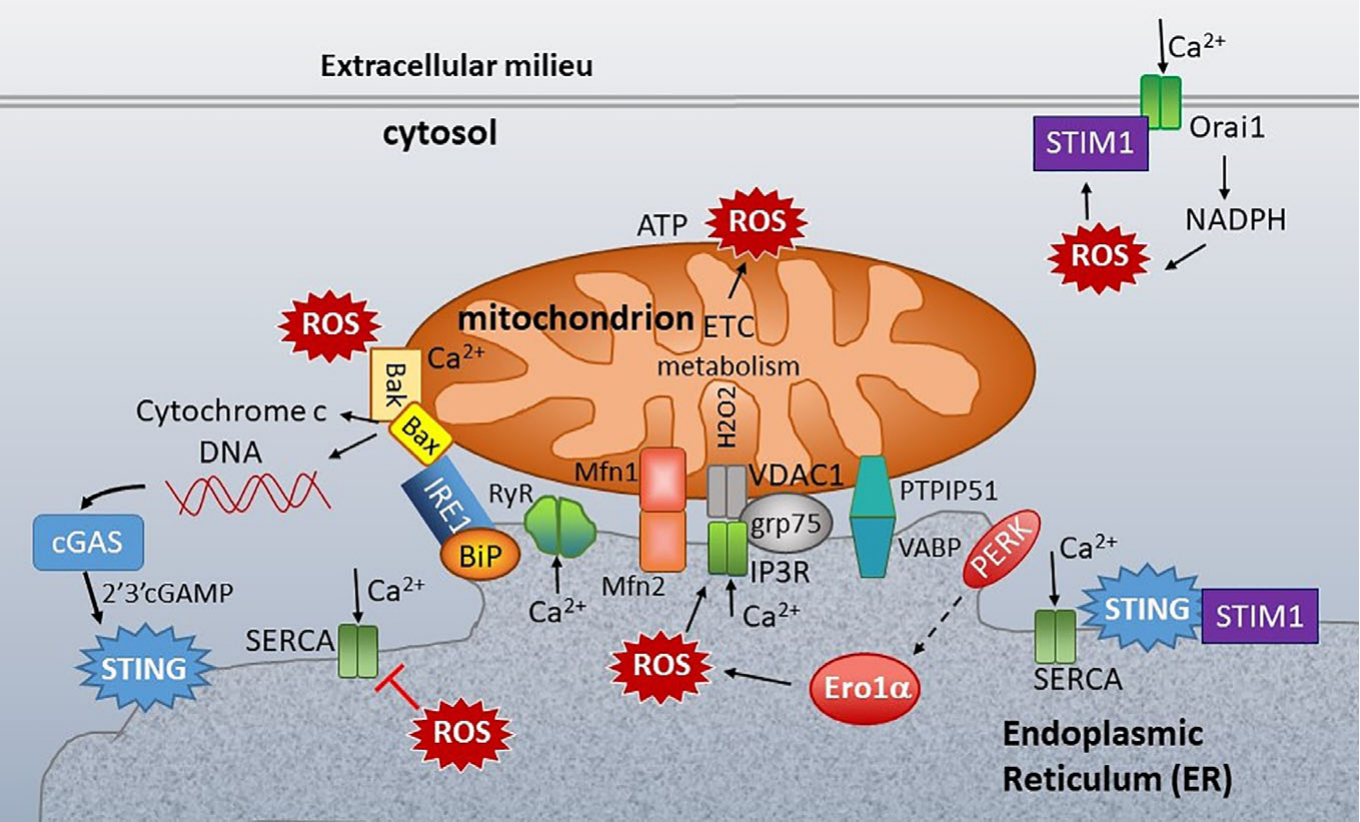
ER-mitochondria connections at the ER mitochondria-associated membranes (MAMs). Mitochondria are closely associated with ER membranes through multiple sets of molecular bridges, including the mitofusins (Mfsn) that regulate mitochondria fission/fusion, the inositol triphosphate receptor (IP3R) calcium channel and non-selective voltage-dependent anion channel (VDAC) stabilized by GRP75, and Vesicle APC-Binding Protein (VAPB) and protein tyrosine phosphatase-interacting protein 51 (PTIP51), which also regulate calcium flux. ER stress sensors inositol-requiring enzyme 1 (IRE1), PKR-like ER kinase (PERK) and folding chaperones (e.g. GRP78/BiP) congregate at the ER mitochondria- associated membranes (MAMs). STING is also enriched at the MAMs. In the resting state, STING associates with STIM1. ER calcium is primarily regulated by three types of calcium channel: Sarcoplasma/ER calcium ATPase (SERCA), which pumps calcium (Ca^2+^) into the ER, and the IP3Rs and ryanodine receptors (RyR), which release ER calcium. Mitochondrial respiration and the action of the electron transport chain (ETC) generate ROS. Protein folding is the primary source of ROS generation in the ER. PERK indirectly induces (dashed arrow) Endoplasmic Reticulum Oxidoreductase 1 Alpha (Ero1α) expression, which is one of the primary sources of ER ROS. ROS decrease ER calcium by inhibiting SERCA and activating IP3R and RyR. ROS also stimulate the translocation of Stromal interaction molecule 1 (STIM1) from ER to plasma membrane, where it interacts with Calcium release-activated calcium channel protein 1 (Orai1) to enable store operated calcium entry (SOC). SOC stimulates NADPH oxidase, generating a positive feedback loop. At the mitochondria, too much calcium and ROS stimulate Bak/Bax mediated release of cytochrome c and extrusion of mitochondrial DNA (mtDNA). The mtDNA stimulates cGAS production of 2’3’-cGAMP, an activating ligand for STING. Calcium regulating molecules are in green, apoptosis in solid yellow, and UPR-associated molecules as in **Figure 5**.

It is evident how mtDNA could stimulate STING *via* cGAS. However, it is much less apparent how ER stress or calcium mechanistically stimulates STING without an activating ligand. This conundrum brings us to the base of our conceptual tripod (**Figure 1**): the ER-mitochondria connection. The ER and mitochondria share a close relationship, both anatomically in the MAMs and functionally. Mitochondria host metabolic pathways, biosynthetic activities, ATP generation, and buffer calcium, generate most of the cellular reactive oxygen species (ROS), and regulate cell death by apoptosis. The ER synthesizes lipids and steroids, regulates calcium, and through oxidative protein folding, generates ROS. We hypothesize that the close connection and functional feedback between these organelles may generate the “missing ligand” in the form of released mitochondrial DNA. Below we will review their interconnections (**Figure 7**) focusing on two “coins of the realm”, calcium (touched on above) and reactive oxygen species (ROS).

Close apposition between ER membranes and mitochondria at the MAMs or MERCs (mitochondria ER contacts) enables phospholipid transfer, calcium movement, and redox control and regulates mitochondrial fusion and fission, inflammasome activation, autophagy, and apoptosis (96, 194). Consider phospholipid synthesis as a prime example of the ER mitochondrial partnership: ER synthesized phosphatidylserine goes to the mitochondria where it is decarboxylated to phosphatidylethanolamine, which then returns to the ER to be methylated to phosphatidylcholine, the most common lipid in cell membranes (195). During mitochondrial fission, the ER first wraps around mitochondria (196). Constriction of the mitochondria *via* ER-bound inverted formin 2 (INF2) requires actin polymerization and increased ER-mitochondria calcium transfer (197). Protein folding requires abundant ATP generated by mitochondrial respiration.

Multiple molecular interactions anatomically bridge the two organelles, facilitating the exchange of small molecules and calcium. These interacting partners include mitofusin2 (Mfn2) on the ER and mitofusin1 (Mfn1) on mitochondria, Vesicle associated membrane protein B (VAPB) and Protein tyrosine phosphatase interacting protein 51 (PTPIP51), IP3R3 and VDAC1 (voltage dependent anion channel 1) ((198–200), **Figure 7**). Mitofusins not only controls ER structure, but also regulate mitophagy and facilitate calcium transfer. VABP and PTPIP51 also facilitate calcium transfer between ER and mitochondria and regulate autophagy (199). On the mitochondrial side, the outer mitochondrial membrane is permeable to calcium through the VDAC channels, but the inner mitochondrial membrane is much less so. However, the steep negative membrane potential generated by respiration can drive the mitochondrial calcium uniporter (MCU) (201). On the external surface, VDACs form pores that allow release of small molecules such as ATP, metabolites, superoxide anions, and cytochrome c. GRP75 stabilizes the bridge between VDACs and IP3Rs (200). Therefore, when the ER releases calcium, particularly through IP3R, the mitochondria are well situated to act as a calcium buffer, maintaining optimally low cytoplasmic calcium. Too much ER calcium release, though and the mitochondria initiate apoptosis. ER stress sensors (more on this below) and protein-folding chaperones also cluster at the MAMs, including BiP, calnexin, calreticulin, ERp44, ERp57, and Sigma 1 receptor (202, 203).

Both ER and mitochondria generate ROS during normal physiologic function and pathologic intracellular stress. ROS also play a critical role in immunity, for instance in NF-κB induction, macrophage phagocytic function and inflammasome activation (204–206). The ER accounts for about 25% of total cellular ROS, primarily produced during protein folding (207). Formation of the inter- and intra-molecular disulfide bonds required for protein structure requires an oxidizing milieu. The Protein disulfide isomerase (PDI) oxidoreductase catalyzes the formation, reduction and isomerization of disulfide bonds. PDI family members ERp57 and ERp72 also form disulfide bonds (208). In order to re-oxidize PDI, electrons are transferred to Endoplasmic Reticulum Oxidoreductase 1 Alpha (ERO1α) *via* a flavin adenine cofactor, and from there to molecular oxygen, ultimately generating H2O2 (209, 210). During ER stress, ERO1α is one of the key downstream targets induced by PERK (via CHOP) to increase folding capacity (211). Too much ERO1α however causes a hyperoxidizing environment, excessive ROS production and thus induces ER stress (212). NADPH family oxidoreductases NOX2, NOX4 and NOX5 also localize to the ER (213, 214). Other ER oxidoreductases filling a similar role to ERO1 include vitamin K epoxide reductase, quiescin sulfydryl oxidase and peroxiredoxin IV (215). Glutathione peroxidases and GSH help scavenge excess ROS (216). Binding of oxidized glutathione peroxidase to BiP enhances its chaperone activity (217). GSH also reduces disulfide bonds in improperly folded proteins. However, there are relatively low levels of GSH in the ER, predisposing to the oxidizing environment. Ratios of GSH:GSSG are 1:1–3:1 vs. 30–100:1 in the cytosol (218).

Mitochondria generate the lion’s share of ROS in the cell *via* fatty acid beta-oxidation, respiration (electron transport chain (ETC) Complex I and III, cytochrome b5 reductase) and other metabolic enzymes including monoamine oxidase, a-ketoglutarate dehydrogenase, pyruvate dehydrogenase, flavoprotein ubiquinone oxidoreductase (219). A subset of NOX4 localizes to the mitochondria where it regulates mitochondrial bioenergetics (220). Superoxide anions are the primary ROS produced by mitochondria. Mitochondrial ROS are scavenged by super oxide dismutases (SODs), glutaredoxin, glutathione, thioredoxin, glutathione reductases, peroxidase and peroxiredoxins (215, 216). Negative feedback loops also keep ROS generation in check. For instance, mitochondrial ROS stabilize Hypoxia inducible factor 1 alpha (HIF1α), which decreases the Krebs cycle and electron transport chain (ETC) activity and stimulates mitophagy (221–224). Mitochondrial fission and fusion also exhibit cross-regulation with ROS; for instance, reactive oxygen species modulator 1 (ROMO1) decreases ROS production and maintains structural integrity by enhancing OPA1 Mitochondrial Dynamin Like GTPase (OPA1) oligomerization (increasing metabolic function and ROS production). However, excessive ROS inactivates ROMO1, leading to cleavage of OPA1, loss of mitochondrial cristae, mitochondrial fragmentation and decreased respiration (225). As an example of positive feedback, excessive ROS induces translocation of the fission-requiring dynamin protein Drp1, and fission promotes mitochondrial ROS over-production (226, 227).

ROS production and calcium fluxes intercommunicate within organelles and between the ER, cytosol and mitochondria on many different levels (**Figure 7**) [reviewed in (171)]. At steady state, constitutive ER calcium release *via* IP3R supports mitochondrial oxidative phosphorylation (228). However, boosting the Krebs cycle dehydrogenases and activating NO synthase increases ROS. ER calcium release that causes cytoplasmic Ca^2+^ spikes generates a “nanodomain” of mitochondria-generated H2O2, which in turn induces a positive feedback increase in calcium release (229). ROS directly regulate the activity of ER calcium channels. For instance, oxidation of RyRs causes calcium leak, further ROS generation and muscle weakness (230). ROS inhibit SERCA by preventing ATP binding, thus depleting ER calcium and increasing cytosolic calcium. ERO1α highly enriches at the MAMs in normal oxidizing conditions (231). ERO1α activity generates H2O2, which oxidizes IP3Rs and results in increased activity and calcium flux out of the ER (232, 233). In the cytosol, increased calcium efflux *via* IP3R stimulates CAMKII, which then exacerbates the situation by stimulating NOX2-dependent ROS production. NOX2 can also stimulate mitochondrial ROS and mitochondrial superoxide activates NOX2 (234, 235). A stressed ER in need of more ATP for folding could thus communicate *via* ROS and calcium to mitochondria to increase ATP production. However too much cytosolic calcium or excess ROS results in opening of the mitochondrial membrane permeability transition pore, resulting in cytochrome c loss, compromise of ECT function (generating more ROS) and initiation of apoptosis (232). Besides activating ER calcium channels, ROS (hydrogen peroxide) also stimulate translocation of STIM1 and possibly STIM2 to the plasma membrane, increasing cytosolic calcium through SOC entry (236, 237). Here also, there is feed-forward reciprocal regulation: STIM1 and Orai1 calcium channels contribute to ROS generation by NADPH oxidase, and NOX2 drives STIM1-mediated SOC entry (238, 239). Putting these observations together, optimum calcium concentrations and limited release enhances communication between organelles and increases their function (i.e. protein folding and metabolic respiration), but excess ROS production and calcium movement out of the ER into mitochondria or cytosol initiates problematic positive feedback loops that can drive apoptosis.

The UPR further impacts ROS and calcium signaling and is in turn regulated by them. The pharmacologic agent Thapsigargin, a SERCA pump inhibitor, rapidly and potently induces the UPR by depleting ER calcium. Oxidized cholesterol causes inflammatory ER stress in macrophages (240). In skeletal muscle, free fatty acids increase oxidative stress and mitochondrial dysfunction, thus leading to ER stress and autophagy. The Sigma1R, which modulates IP3R activity and calcium flux, decreases ER stress and stabilizes IRE1 oligomerization and generation of pro-survival responses (202, 241). TLR signaling induces IRE1 activation and XBP1 production *via* NOX2 by an unclear mechanism (156). In the direction of ER stress to calcium/ROS, ER stress increases cytosolic calcium to the point of calcium-dependent mitochondrial outer membrane permeabilization and apoptosis. PERK, which is abundant at the MAMs, contributes to ROS-driven mitochondrial stress and apoptosis (242). PERK both increases ROS *via* ERO1α induction and conversely induces anti-oxidant responses *via* nuclear factor erythroid 2–related factor 2 (Nrf2). PERK directly phosphorylates Nrf2, leading to its dissociation from Kelch-like ECH-associated protein 1 (KEAP1) which prevents Nrf2 nuclear translocation (243). With ATF4, Nrf2 induces SODs, Heme oxygenase-1, glutathione transferase and uncoupling mitochondrial protein 2 (UCP2) (215). PERK regulation of proteostasis (and oxidative ROS-generating protein folding) can also have a large impact on cell capacity to survive ER stress (244).

## Evidence for a Triad or Guilt by Association?

To this point, we have addressed the various dyads between STING and mitochondria, STING and ER, and mitochondria and ER, but what evidence is there for a three-way interaction? UPR activation has been previously implicated in some of the same settings where mitochondrial damage is now taking the spotlight. Consider the case of STING and the RNA virus Dengue virus. The elaboration of viral mitochondria-targeting proteins and resulting mitochondrial stress and damage was described above. Dengue replicates in ER-derived vesicles and also induces the UPR. Viral induction of PERK and IRE1 signaling pathways increase viral autophagy and replication (245). Similarly, in the case of Cisplatin induced acute kidney injury, Cisplatin has long been known to cause ER stress and UPR activation (246). In these two scenarios, mtDNA-induced STING activation and UPR coexist in the same pathologic setting, but the whether these manifestations are interconnected or occur independently is not yet clear.

**Figure 8:**
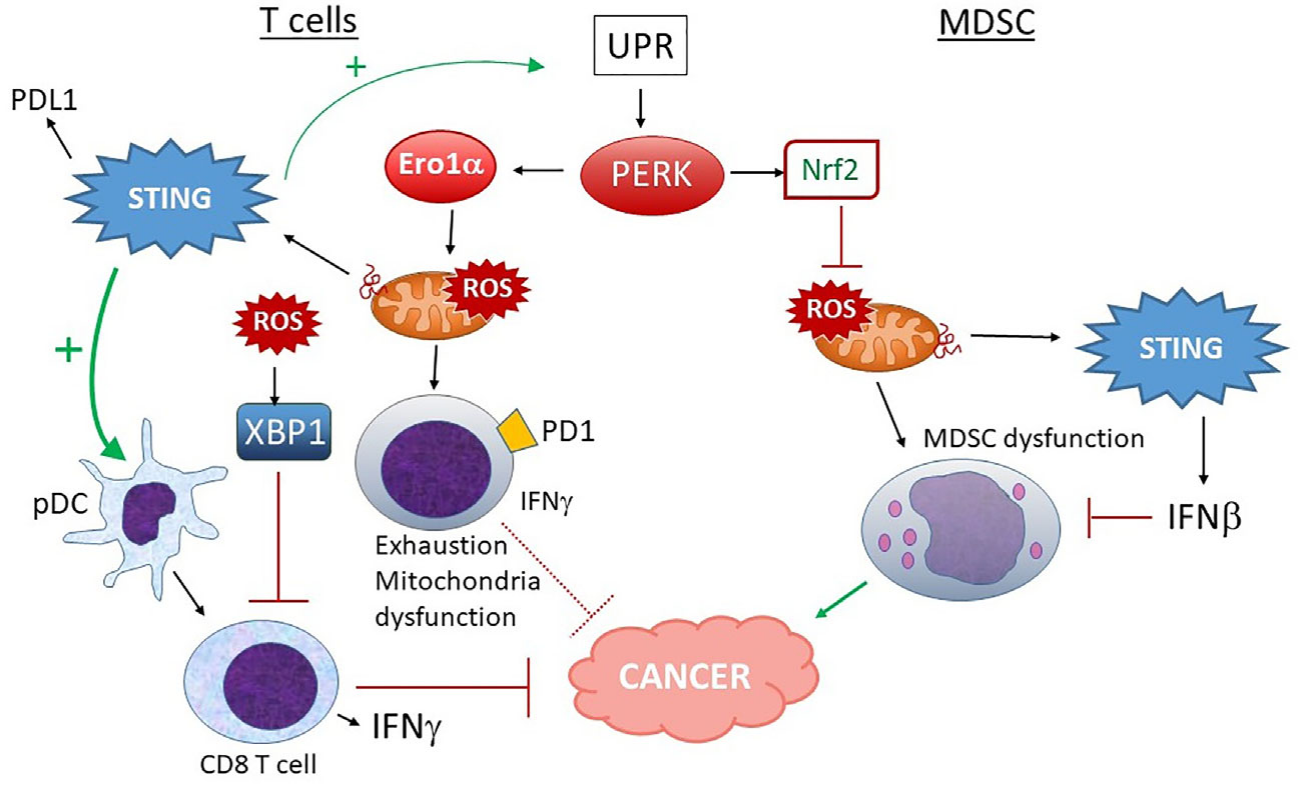
Different outcomes of PERK activation in T cells vs. MDSC and STING input. In T cells, PERK and ERO1α increase ROS production, leading to mitochondrial dysfunction, increased exhaustion and lower IFNγ production, rendering these PD1+ T cells less adept at fighting tumors. ROS-stimulated XBP1 also decreases T cell activation. Although STING activation could make matters worse by increasing UPR induction and PDL1 expression, many studies indicate a positive role for STING and type I IFN in pDC-dependent CD8 T cell activation and anti-tumor activities, suggesting a balance of effects. On the other hand, in MDSC, PERK-stimulated Nrf2 activity predominates. Nrf2 prevents mitochondrial ROS and dysfunction. Mitochondrial ROS also leads to dsDNA extrusion and STING activation, which further inhibit MDSC *via* Type I IFN signaling. MDSC promote tumor progression.

More work on the relationship between UPR and mitochondria had been done in cancer (**Figure 8**). One report suggested ER stress contributes to mitochondrial exhaustion of CD8 T cells (247). In a murine sarcoma model, tumor-infiltrating PD1+ cells had greater levels of mitochondrial ROS. Mitochondrial ROS correlated with mitochondrial dysfunction as evident by lower oxygen consumption rates. PERK inhibition decreased mitochondrial ROS in PD1+ cells. PERK inhibitor and ERO1 inhibitor treated T cells exhibited both higher O2 consumption rates and improved IFN-γ production. IFN-γ, produced by tumor-infiltrating CD8 T cells and NK cells, enhances cytotoxicity and antigen presentation, and exerts direct anti-tumor effects – although in some settings, the cytokine may be pro-tumorigenic (248). Further, PERK deficiency and PERK and ERO1α inhibitor treatment of T cells resulted in higher energy reserve and enhanced anti-tumor activity *in vivo*. Others have shown constitutive XBP1 activation by ROS (lipid peroxidation byproducts more specifically) drives tumor progression by limiting antigen presentation and T cell activation (249). Thus, through IRE1 or PERK, the UPR can have a deleterious effect on cancer containment. However, STING agonists (which can increase ER stress) improve CD8 T cell anti-tumor activity, despite increasing PDL1 expression (49). When it comes to T cell regulation, STING may exert competing effects on IFN production vs. ER stress and exhaustion. Thus, in developing therapeutics, the various effects of STING agonists on mitochondria and UPR signaling in CD8 T cells or their interacting DCs will require clarification in specific contexts.

The effects of UPR/PERK-mitochondria signaling may also be cell-specific. Myeloid derived suppressor cells (MDSC) have been implicated in tumor progression. These cells show signs of UPR activation correlating with chemoresistance (250). Thapsigargin treatment expanded splenic MDSC and enhanced tumor growth whereas the UPR inhibitor TUDCA had the opposite effect. Mohamed et al. reported that the PERK pathway was highly activated in MDSC in tumors (251). PERK enhanced MDSC-mediated immunosuppression *via* Nrf2, preventing oxidative damage, mitochondrial DNA release and DNA sensing *via* cGAS/STING. Ablating PERK in the myeloid branch delayed tumor growth. In a separate report, CHOP contributed to MDSC activity. However, the PERK effect noted by Mohamed et al. required Nrf2, not CHOP. PERK deficient MDSC exhibited increased cellular ROS, altered mitochondrial morphology, membrane potential, reduced oxygen consumption and release of mtDNA. The mtDNA activated STING and induced type I IFN. Blocking STING or IFNAR restored the immunosuppressive effect of MDSC in the absence of PERK. Thus, in the case of MDSC, PERK promoted suppressor cell “well-being” and inhibited STING activation through its anti-oxidant activities (251). Interestingly, Nrf2 also antagonizes STING expression by mRNA destabilization (252).

Thus, in some settings STING agonists hold dramatic promise as anti-tumor agents. However, other reports suggest they may increase metastases and tumor progression. We are just beginning to scratch the surface of how STING regulates different types of tumors and the different cells in tumor environments. Understanding the potential mechanisms by which ER stress and mitochondrial dysfunction interact and regulate STING is lagging further behind but a ripe area for further study.

## Summary and Perspective

An underlying hypothesis in this review is that ER stress may activate STING in the absence of an obvious ligand *via* calcium/ROS mediated mitochondrial damage and mtDNA release. To illustrate how this might work based on the previous discussion consider cancer once more: In tumor microenvironments, unregulated cellular proliferation may outstrip the neo-vascular supply of nutrients including oxygen, glucose and amino acids. This lack of nutrients negatively impacts ER function, triggering the UPR. Hypoxia may directly uncouple electron transport and damage mitochondria. However, it is also likely that the disruptions in ER calcium homeostasis, ROS production and stress will lead to mitochondrial damage and release of mtDNA into the cytosol. cGAS would then sense the mtDNA and generate cGAMP, which stimulates STING to produce type I IFN. This scenario raises multiple questions: It may be a logical fallacy to invoke crosstalk between all three corners of the triad; just because A goes to B and B goes to C, doesn’t mean A requires B to get to C. The effect of hypoxia on mitochondria may be sufficient in the absence of ER stress to cause mtDNA release. ER stress may activate STING in some unknown way without the mitochondrial intermediary, for instance by stabilizing STING oligomerization or altering STING trafficking.

The data presented above raise other questions regarding ER stress-mitochondria-STING interactions: It is still unclear why calcium mobilization during ER stress was necessary for Thapsigargin and oxygen-glucose induced STING activation—was it because of a unique calcium-dependent effect on mitochondria and subsequent mitochondrial DNA release or another mechanism? Was the role of the ER stress simply to generate ROS? Another issue is how the UPR could trigger mtDNA release without initiating apoptosis. The UPR triggers multiple pathways converging on mitochondria-dependent intrinsic apoptosis including suppression of anti-apoptotic molecules, induction of pro-apoptotic molecules and JNK signaling, in addition to the calcium and ROS dysregulation described above (253). The decision between mitochondrial DNA release and apoptosis may simply be a matter of degree of ER stress and relative amount of mitochondrial destruction, but such a threshold model would require further experimental support. There is certainly evidence for a yin-yang balance between apoptosis and mtDNA stimulation of STING in that caspase deficiency increases STING induced IFN (254). Alternatively, in addition to Bax-Bak pores, VDAC pores in oxidatively stressed mitochondria enable mtDNA extrusion, perhaps promoting STING activation short of apoptosis (255).

Let us come back full circle. What is the physiologic need for repurposing PRRs such as STING? One possibility is the context added by DAMPs; inside the cells, sufficient damage from pathogens can trigger PRRs to amplify immune responses. However, endogenous PRR stimulation represents a double-edged sword with its own perils, as manifest by the involvement of STING in heart disease, cancer and autoimmunity. In cancer, STING stimulation by endogenous stressors not only can bolster innate and adaptive anti-tumor immunity but can also undermine anti-tumor defenses. Similarly, STING may drive type I interferonapathies, but STING deficiency exacerbates autoimmunity triggered by other PRRs. STING is particularly well situated to respond to organelle-generated alarm signals resulting from disruptions in calcium homeostasis and critically increased reactive oxygen species. The close apposition of ER and mitochondria and calcium-ROS cross talk between these organelles offers the tantalizing possibility that stress initiated in either organelle could ultimately generate the required ligand for STING and regulate STING activity. It will be interesting to see how elucidation of the underlying mechanisms leading from intracellular stress and damage to STING activation unfolds. Linear sequential pathways are much easier to assess *via* common tools such as expression modulation or inhibitors, but reciprocal regulation and mutually augmenting feedback loops present much more of a challenge. Despite these issues, it remains important to determine the key intermediaries and interactions within these pathways under different scenarios, because this knowledge will be critical for guiding therapeutic interventions.

## Author Contributions

The author confirms being the sole contributor of this work and has approved it for publication.

## Funding

All funding for this work comes from the University of Wisconsin-Madison (institutional support).

## Conflict of Interest

The author declares that the research was conducted in the absence of any commercial or financial relationships that could be construed as a potential conflict of interest.

## References

[1] Bianchi ME DAMPs, PAMPs and alarmins: all we need to know about danger. J Leukoc Biol (2007) 81(1):1–5. 10.1189/jlb.0306164 17032697

[2] Mollen KP Anand RJ Tsung A Prince JM Levy RM Billiar TR Emerging paradigm: toll-like receptor 4-sentinel for the detection of tissue damage. Shock (2006) 26(5):430–7. 10.1097/01.shk.0000228797.41044.08 17047512

[3] Yu L Wang L Chen S Endogenous toll-like receptor ligands and their biological significance. J Cell Mol Med (2010) 14(11):2592–603. 10.1111/j.1582-4934.2010.01127.x PMC4373479 20629986

[4] Anthoney N Foldi I Hidalgo A Toll and Toll-like receptor signalling in development. Development (2018) 145(9):dev156018. 10.1242/dev.156018 29695493

[5] Chen CY Shih YC Hung YF Hsueh YP Beyond defense: regulation of neuronal morphogenesis and brain functions via Toll-like receptors. J BioMed Sci (2019) 26(1):90. 10.1186/s12929-019-0584-z 31684953 PMC6827257

[6] Tilstra JS John S Gordon RA Leibler C Kashgarian M Bastacky S B cell-intrinsic TLR9 expression is protective in murine lupus. J Clin Invest (2020) 130(6):3172–87. 10.1172/JCI132328 PMC7260024 32191633

[7] Ewald SE Barton GM Nucleic acid sensing Toll-like receptors in autoimmunity. Curr Opin Immunol (2011) 23(1):3–9. 10.1016/j.coi.2010.11.006 21146971 PMC3057394

[8] Ishikawa H Ma Z Barber GN STING regulates intracellular DNA-mediated, type I interferon-dependent innate immunity. Nature (2009) 461(7265):788–92. 10.1038/nature08476 PMC4664154 19776740

[9] Mohanty A Tiwari-Pandey R Pandey NR Mitochondria: the indispensable players in innate immunity and guardians of the inflammatory response. J Cell Commun Signal (2019) 13(3):303–18. 10.1007/s12079-019-00507-9 PMC6732146 30719617

[10] Seth RB Sun L Ea CK Chen ZJ Identification and characterization of MAVS, a mitochondrial antiviral signaling protein that activates NF-kappaB and IRF 3. Cell (2005) 122(5):669–82. 10.1016/j.cell.2005.08.012 16125763

[11] Wu B Hur S How RIG-I like receptors activate MAVS. Curr Opin Virol (2015) 12:91–8. 10.1016/j.coviro.2015.04.004 PMC4470786 25942693

[12] Kranzusch PJ Wilson SC Lee AS Berger JM Doudna JA Vance RE Ancient Origin of cGAS-STING Reveals Mechanism of Universal 2’,3’ cGAMP Signaling. Mol Cell (2015) 59(6):891–903. 10.1016/j.molcel.2015.07.022 26300263 PMC4575873

[13] Margolis SR Wilson SC Vance RE Evolutionary Origins of cGAS-STING Signaling. Trends Immunol (2017) 38(10):733–43. 10.1016/j.it.2017.03.004 28416447

[14] Sun L Wu J Du F Chen X Chen ZJ Cyclic GMP-AMP synthase is a cytosolic DNA sensor that activates the type I interferon pathway. Science (2013) 339(6121):786–91. 10.1126/science.1232458 PMC3863629 23258413

[15] Li XD Wu J Gao D Wang H Sun L Chen ZJ Pivotal roles of cGAS-cGAMP signaling in antiviral defense and immune adjuvant effects. Science (2013) 341(6152):1390–4. 10.1126/science.1244040 PMC3863637 23989956

[16] Jonsson KL Laustsen A Krapp C Skipper KA Thavachelvam K Hotter D IFI16 is required for DNA sensing in human macrophages by promoting production and function of cGAMP. Nat Commun (2017) 8:14391. 10.1038/ncomms14391 28186168 PMC5309897

[17] Almine JF O’Hare CA Dunphy G Haga IR Naik RJ Atrih A IFI16 and cGAS cooperate in the activation of STING during DNA sensing in human keratinocytes. Nat Commun (2017) 8:14392. 10.1038/ncomms14392 28194029 PMC5316833

[18] Ma F Li B Liu SY Iyer SS Yu Y Wu A Positive feedback regulation of type I IFN production by the IFN-inducible DNA sensor cGAS. J Immunol (2015) 194(4):1545–54. 10.4049/jimmunol.1402066 PMC4324085 25609843

[19] Gentili M Lahaye X Nadalin F Nader GPF Lombardi EP Herve S The N-Terminal Domain of cGAS Determines Preferential Association with Centromeric DNA and Innate Immune Activation in the Nucleus. Cell Rep (2019) 26(13):3798. 10.1016/j.celrep.2019.03.049 30917330 PMC6444014

[20] Orzalli MH Broekema NM Diner BA Hancks DC Elde NC Cristea IM cGAS-mediated stabilization of IFI16 promotes innate signaling during herpes simplex virus infection. Proc Natl Acad Sci U S A (2015) 112(14):E1773–81. 10.1073/pnas.1424637112 PMC4394261 25831530

[21] Volkman HE Cambier S Gray EE Stetson DB Tight nuclear tethering of cGAS is essential for preventing autoreactivity. eLife (2019) 8:e47491. 10.7554/eLife.47491 31808743 PMC6927687

[22] Barnett KC Coronas-Serna JM Zhou W Ernandes MJ Cao A Kranzusch PJ Phosphoinositide Interactions Position cGAS at the Plasma Membrane to Ensure Efficient Distinction between Self- and Viral DNA. Cell (2019) 176(6):1432–46.e11. 10.1016/j.cell.2019.01.049 30827685 PMC6697112

[23] Michalski S de Oliveira Mann CC Stafford C Witte G Bartho J Lammens K Structural basis for sequestration and autoinhibition of cGAS by chromatin. Nature (2020) 587(7835):678–82. 10.1038/s41586-020-2748-0 32911480

[24] Zhao B Xu P Rowlett CM Jing T Shinde O Lei Y The Molecular Basis of Tight Nuclear Tethering and Inactivation of cGAS. Nature (2020) 587(7835):673–7. 10.1038/s41586-020-2749-z PMC7704945 32911481

[25] Pathare GR Decout A Gluck S Cavadini S Makasheva K Hovius R Structural mechanism of cGAS inhibition by the nucleosome. Nature (2020) 587(7835):668–72. 10.1038/s41586-020-2750-6 32911482

[26] Boyer JA Spangler CJ Strauss JD Cesmat AP Liu P McGinty RK Structural basis of nucleosome-dependent cGAS inhibition. Science (2020) 370(6515):450–4. 10.1126/science.abd0609 PMC8189757 32913000

[27] Cao D Han X Fan X Xu RM Zhang X Structural basis for nucleosome-mediated inhibition of cGAS activity. Cell Res (2020) 30(12):1088–97. 10.1038/s41422-020-00422-4 PMC7784699 33051594

[28] Lahaye X Gentili M Silvin A Conrad C Picard L Jouve M NONO Detects the Nuclear HIV Capsid to Promote cGAS-Mediated Innate Immune Activation. Cell (2018) 175(2):488–501.e22. 10.1016/j.cell.2018.08.062 30270045

[29] Herzner AM Hagmann CA Goldeck M Wolter S Kubler K Wittmann S Sequence-specific activation of the DNA sensor cGAS by Y-form DNA structures as found in primary HIV-1 cDNA. Nat Immunol (2015) 16(10):1025–33. 10.1038/ni.3267 PMC4669199 26343537

[30] Gao D Wu J Wu YT Du F Aroh C Yan N Cyclic GMP-AMP synthase is an innate immune sensor of HIV and other retroviruses. Science (2013) 341(6148):903–6. 10.1126/science.1240933 PMC3860819 23929945

[31] Luecke S Holleufer A Christensen MH Jonsson KL Boni GA Sorensen LK cGAS is activated by DNA in a length-dependent manner. EMBO Rep (2017) 18(10):1707–15. 10.15252/embr.201744017 PMC5623850 28801534

[32] Nelson PN Carnegie PR Martin J Davari Ejtehadi H Hooley P Roden D Demystified. Human endogenous retroviruses. Mol Pathol (2003) 56(1):11–8. 10.1136/mp.56.1.11 PMC1187282 12560456

[33] Civril F Deimling T de Oliveira Mann CC Ablasser A Moldt M Witte G Structural mechanism of cytosolic DNA sensing by cGAS. Nature (2013) 498(7454):332–7. 10.1038/nature12305 PMC3768140 23722159

[34] Du M Chen ZJ DNA-induced liquid phase condensation of cGAS activates innate immune signaling. Science (2018) 361(6403):704–9. 10.1126/science.aat1022 PMC9417938 29976794

[35] Li X Shu C Yi G Chaton CT Shelton CL Diao J Cyclic GMP-AMP synthase is activated by double-stranded DNA-induced oligomerization. Immunity (2013) 39(6):1019–31. 10.1016/j.immuni.2013.10.019 PMC3886715 24332030

[36] Gao P Ascano M Wu Y Barchet W Gaffney BL Zillinger T Cyclic [G(2’,5’)pA(3’,5’)p] is the metazoan second messenger produced by DNA-activated cyclic GMP-AMP synthase. Cell (2013) 153(5):1094–107. 10.1016/j.cell.2013.04.046 PMC4382009 23647843

[37] Zhang X Shi H Wu J Zhang X Sun L Chen C Cyclic GMP-AMP containing mixed phosphodiester linkages is an endogenous high-affinity ligand for STING. Mol Cell (2013) 51(2):226–35. 10.1016/j.molcel.2013.05.022 PMC3808999 23747010

[38] Burdette DL Monroe KM Sotelo-Troha K Iwig JS Eckert B Hyodo M STING is a direct innate immune sensor of cyclic di-GMP. Nature (2011) 478(7370):515–8. 10.1038/nature10429 PMC3203314 21947006

[39] Woodward JJ Iavarone AT Portnoy DA c-di-AMP secreted by intracellular Listeria monocytogenes activates a host type I interferon response. Science (2010) 328(5986):1703–5. 10.1126/science.1189801 PMC3156580 20508090

[40] Sauer JD Sotelo-Troha K von Moltke J Monroe KM Rae CS Brubaker SW The N-ethyl-N-nitrosourea-induced Goldenticket mouse mutant reveals an essential function of Sting in the in vivo interferon response to Listeria monocytogenes and cyclic dinucleotides. Infect Immun (2011) 79(2):688–94. 10.1128/IAI.00999-10 PMC3028833 21098106

[41] Gao P Ascano M Zillinger T Wang W Dai P Serganov AA Structure-function analysis of STING activation by c[G(2’,5’)pA(3’,5’)p] and targeting by antiviral DMXAA. Cell (2013) 154(4):748–62. 10.1016/j.cell.2013.07.023 PMC4386733 23910378

[42] McFarland AP Luo S Ahmed-Qadri F Zuck M Thayer EF Goo YA Sensing of Bacterial Cyclic Dinucleotides by the Oxidoreductase RECON Promotes NF-kappaB Activation and Shapes a Proinflammatory Antibacterial State. Immunity (2017) 46(3):433–45. 10.1016/j.immuni.2017.02.014 PMC5404390 28329705

[43] Auerbuch V Brockstedt DG Meyer-Morse N O’Riordan M Portnoy DA Mice lacking the type I interferon receptor are resistant to Listeria monocytogenes. J Exp Med (2004) 200(4):527–33. 10.1084/jem.20040976 PMC2211930 15302899

[44] de Almeida LA Carvalho NB Oliveira FS Lacerda TL Vasconcelos AC Nogueira L MyD88 and STING signaling pathways are required for IRF3-mediated IFN-beta induction in response to Brucella abortus infection. PLoS One (2011) 6(8):e23135. 10.1371/journal.pone.0023135 21829705 PMC3149075

[45] Burdette DL Vance RE STING and the innate immune response to nucleic acids in the cytosol. Nat Immunol (2013) 14(1):19–26. 10.1038/ni.2491 23238760

[46] Shang G Zhang C Chen ZJ Bai XC Zhang X Cryo-EM structures of STING reveal its mechanism of activation by cyclic GMP-AMP. Nature (2019) 567(7748):389–93. 10.1038/s41586-019-0998-5 PMC6859894 30842659

[47] Zhao B Du F Xu P Shu C Sankaran B Bell SL A conserved PLPLRT/SD motif of STING mediates the recruitment and activation of TBK1. Nature (2019) 569(7758):718–22. 10.1038/s41586-019-1228-x PMC6596994 31118511

[48] Ergun SL Fernandez D Weiss TM Li L STING Polymer Structure Reveals Mechanisms for Activation, Hyperactivation, and Inhibition. Cell (2019) 178(2):290–301. 10.1016/j.cell.2019.05.036 31230712

[49] Chin EN Yu C Vartabedian VF Jia Y Kumar M Gamo AM Antitumor activity of a systemic STING-activating non-nucleotide cGAMP mimetic. Science (2020) 369(6506):993–9. 10.1126/science.abb4255 32820126

[50] Balka KR Louis C Saunders TL Smith AM Calleja DJ D’Silva DB TBK1 and IKKepsilon Act Redundantly to Mediate STING-Induced NF-kappaB Responses in Myeloid Cells. Cell Rep (2020) 31(1):107492. 10.1016/j.celrep.2020.03.056 32268090

[51] Zhang C Shang G Gui X Zhang X Bai XC Chen ZJ Structural basis of STING binding with and phosphorylation by TBK1. Nature (2019) 567(7748):394–8. 10.1038/s41586-019-1000-2 PMC6862768 30842653

[52] Liu S Cai X Wu J Cong Q Chen X Li T Phosphorylation of innate immune adaptor proteins MAVS, STING, and TRIF induces IRF3 activation. Science (2015) 347(6227):aaa2630. 10.1126/science.aaa2630 25636800

[53] Hiscott J Pitha P Genin P Nguyen H Heylbroeck C Mamane Y Triggering the interferon response: the role of IRF-3 transcription factor. J Interferon Cytokine Res (1999) 19(1):1–13. 10.1089/107999099314360 10048763

[54] Gui X Yang H Li T Tan X Shi P Li M Autophagy induction via STING trafficking is a primordial function of the cGAS pathway. Nature (2019) 567(7747):262–6. 10.1038/s41586-019-1006-9 PMC9417302 30842662

[55] Ogawa E Mukai K Saito K Arai H Taguchi T The binding of TBK1 to STING requires exocytic membrane traffic from the ER. Biochem Biophys Res Commun (2018) 503(1):138–45. 10.1016/j.bbrc.2018.05.199 29870684

[56] Dobbs N Burnaevskiy N Chen D Gonugunta VK Alto NM Yan N STING Activation by Translocation from the ER Is Associated with Infection and Autoinflammatory Disease. Cell Host Microbe (2015) 18(2):157–68. 10.1016/j.chom.2015.07.001 PMC4537353 26235147

[57] Chen H Sun H You F Sun W Zhou X Chen L Activation of STAT6 by STING is critical for antiviral innate immunity. Cell (2011) 147(2):436–46. 10.1016/j.cell.2011.09.022 22000020

[58] Abe T Barber GN Cytosolic-DNA-mediated, STING-dependent proinflammatory gene induction necessitates canonical NF-kappaB activation through TBK1. J Virol (2014) 88(10):5328–41. 10.1128/JVI.00037-14 PMC4019140 24600004

[59] Saitoh T Fujita N Hayashi T Takahara K Satoh T Lee H Atg9a controls dsDNA-driven dynamic translocation of STING and the innate immune response. Proc Natl Acad Sci U S A (2009) 106(49):20842–6. 10.1073/pnas.0911267106 PMC2791563 19926846

[60] Gulen MF Koch U Haag SM Schuler F Apetoh L Villunger A Signalling strength determines proapoptotic functions of STING. Nat Commun (2017) 8(1):427. 10.1038/s41467-017-00573-w 28874664 PMC5585373

[61] Gaidt MM Ebert TS Chauhan D Ramshorn K Pinci F Zuber S The DNA Inflammasome in Human Myeloid Cells Is Initiated by a STING-Cell Death Program Upstream of NLRP3. Cell (2017) 171(5):1110–24.e18. 10.1016/j.cell.2017.09.039 29033128 PMC5901709

[62] Swanson KV Junkins RD Kurkjian CJ Holley-Guthrie E Pendse AA El Morabiti R A noncanonical function of cGAMP in inflammasome priming and activation. J Exp Med (2017) 214(12):3611–26. 10.1084/jem.20171749 PMC5716045 29030458

[63] Franz KM Neidermyer WJ Tan YJ Whelan SPJ Kagan JC STING-dependent translation inhibition restricts RNA virus replication. Proc Natl Acad Sci U S A (2018) 115(9):E2058–E67. 10.1073/pnas.1716937115 PMC5834695 29440426

[64] Yamashiro LH Wilson SC Morrison HM Karalis V Chung JJ Chen KJ Interferon-independent STING signaling promotes resistance to HSV-1 in vivo. Nat Commun (2020) 11(1):3382. 10.1038/s41467-020-17156-x 32636381 PMC7341812

[65] Chen H Pei R Zhu W Zeng R Wang Y Wang Y An alternative splicing isoform of MITA antagonizes MITA-mediated induction of type I IFNs. J Immunol (2014) 192(3):1162–70. 10.4049/jimmunol.1300798 24391220

[66] Dunphy G Flannery SM Almine JF Connolly DJ Paulus C Jonsson KL Non-canonical Activation of the DNA Sensing Adaptor STING by ATM and IFI16 Mediates NF-kappaB Signaling after Nuclear DNA Damage. Mol Cell (2018) 71(5):745–60. 10.1016/j.molcel.2018.07.034 PMC6127031 30193098

[67] de Oliveira Mann CC Orzalli MH King DS Kagan JC Lee ASY Kranzusch PJ Modular Architecture of the STING C-Terminal Tail Allows Interferon and NF-kappaB Signaling Adaptation. Cell Rep (2019) 27(4):1165–75. 10.1016/j.celrep.2019.03.098 PMC7733315 31018131

[68] Wu X Wu FH Wang X Wang L Siedow JN Zhang W Molecular evolutionary and structural analysis of the cytosolic DNA sensor cGAS and STING. Nucleic Acids Res (2014) 42(13):8243–57. 10.1093/nar/gku569 PMC4117786 24981511

[69] Richter B Sliter DA Herhaus L Stolz A Wang C Beli P Phosphorylation of OPTN by TBK1 enhances its binding to Ub chains and promotes selective autophagy of damaged mitochondria. Proc Natl Acad Sci U S A (2016) 113(15):4039–44. 10.1073/pnas.1523926113 PMC4839414 27035970

[70] Liang Q Seo GJ Choi YJ Kwak MJ Ge J Rodgers MA Crosstalk between the cGAS DNA sensor and Beclin-1 autophagy protein shapes innate antimicrobial immune responses. Cell Host Microbe (2014) 15(2):228–38. 10.1016/j.chom.2014.01.009 PMC3950946 24528868

[71] Murthy AMV Robinson N Kumar S Crosstalk between cGAS-STING signaling and cell death. Cell Death Differ (2020) 27(11):2989–3003. 10.1038/s41418-020-00624-8 32948836 PMC7560597

[72] Westphal D Kluck RM Dewson G Building blocks of the apoptotic pore: how Bax and Bak are activated and oligomerize during apoptosis. Cell Death Differ (2014) 21(2):196–205. 10.1038/cdd.2013.139 24162660 PMC3890949

[73] McArthur K Whitehead LW Heddleston JM Li L Padman BS Oorschot V BAK/BAX macropores facilitate mitochondrial herniation and mtDNA efflux during apoptosis. Science (2018) 359(6378):eaa06047. 10.1126/science.aao6047 29472455

[74] Chen D Tong J Yang L Wei L Stolz DB Yu J PUMA amplifies necroptosis signaling by activating cytosolic DNA sensors. Proc Natl Acad Sci U S A (2018) 115(15):3930–5. 10.1073/pnas.1717190115 PMC5899441 29581256

[75] Chattopadhyay S Marques JT Yamashita M Peters KL Smith K Desai A Viral apoptosis is induced by IRF-3-mediated activation of Bax. EMBO J (2010) 29(10):1762–73. 10.1038/emboj.2010.50 PMC2876960 20360684

[76] Brault M Olsen TM Martinez J Stetson DB Oberst A Intracellular Nucleic Acid Sensing Triggers Necroptosis through Synergistic Type I IFN and TNF Signaling. J Immunol (2018) 200(8):2748–56. 10.4049/jimmunol.1701492 PMC5893403 29540580

[77] Sarhan J Liu BC Muendlein HI Weindel CG Smirnova I Tang AY Constitutive interferon signaling maintains critical threshold of MLKL expression to license necroptosis. Cell Death Differ (2019) 26(2):332–47. 10.1038/s41418-018-0122-7 PMC6329789 29786074

[78] Miao EA Leaf IA Treuting PM Mao DP Dors M Sarkar A Caspase-1-induced pyroptosis is an innate immune effector mechanism against intracellular bacteria. Nat Immunol (2010) 11(12):1136–42. 10.1038/ni.1960 PMC3058225 21057511

[79] Hu MM Shu HB Innate Immune Response to Cytoplasmic DNA: Mechanisms and Diseases. Annu Rev Immunol (2020) 38:79–98. 10.1146/annurev-immunol-070119-115052 31800327

[80] Wan D Jiang W Hao J Research Advances in How the cGAS-STING Pathway Controls the Cellular Inflammatory Response. Front Immunol (2020) 11:615. 10.3389/fimmu.2020.00615 32411126 PMC7198750

[81] Hopfner KP Hornung V Molecular mechanisms and cellular functions of cGAS-STING signalling. Nat Rev Mol Cell Biol (2020) 21(9):501–21. 10.1038/s41580-020-0244-x 32424334

[82] Ma F Li B Yu Y Iyer SS Sun M Cheng G Positive feedback regulation of type I interferon by the interferon-stimulated gene STING. EMBO Rep (2015) 16(2):202–12. 10.15252/embr.201439366 PMC4328747 25572843

[83] Xia T Konno H Ahn J Barber GN Deregulation of STING Signaling in Colorectal Carcinoma Constrains DNA Damage Responses and Correlates With Tumorigenesis. Cell Rep (2016) 14(2):282–97. 10.1016/j.celrep.2015.12.029 PMC4845097 26748708

[84] Mukai K Konno H Akiba T Uemura T Waguri S Kobayashi T Activation of STING requires palmitoylation at the Golgi. Nat Commun (2016) 7:11932. 10.1038/ncomms11932 27324217 PMC4919521

[85] Xia P Ye B Wang S Zhu X Du Y Xiong Z Glutamylation of the DNA sensor cGAS regulates its binding and synthase activity in antiviral immunity. Nat Immunol (2016) 17(4):369–78. 10.1038/ni.3356 26829768

[86] Seo GJ Yang A Tan B Kim S Liang Q Choi Y Akt Kinase-Mediated Checkpoint of cGAS DNA Sensing Pathway. Cell Rep (2015) 13(2):440–9. 10.1016/j.celrep.2015.09.007 PMC4607670 26440888

[87] Konno H Konno K Barber GN Cyclic dinucleotides trigger ULK1 (ATG1) phosphorylation of STING to prevent sustained innate immune signaling. Cell (2013) 155(3):688–98. 10.1016/j.cell.2013.09.049 PMC3881181 24119841

[88] Ning X Wang Y Jing M Sha M Lv M Gao P Apoptotic Caspases Suppress Type I Interferon Production via the Cleavage of cGAS, MAVS, and IRF3. Mol Cell (2019) 74(1):19–31.e7. 10.1016/j.molcel.2019.02.013 30878284

[89] White MJ McArthur K Metcalf D Lane RM Cambier JC Herold MJ Apoptotic caspases suppress mtDNA-induced STING-mediated type I IFN production. Cell (2014) 159(7):1549–62. 10.1016/j.cell.2014.11.036 PMC4520319 25525874

[90] Wang Y Ning X Gao P Wu S Sha M Lv M Inflammasome Activation Triggers Caspase-1-Mediated Cleavage of cGAS to Regulate Responses to DNA Virus Infection. Immunity (2017) 46(3):393–404. 10.1016/j.immuni.2017.02.011 28314590

[91] Banerjee I Behl B Mendonca M Shrivastava G Russo AJ Menoret A Gasdermin D Restrains Type I Interferon Response to Cytosolic DNA by Disrupting Ionic Homeostasis. Immunity (2018) 49(3):413–26.e5. 10.1016/j.immuni.2018.07.006 30170814 PMC6347470

[92] Corrales L McWhirter SM Dubensky TW Jr. Gajewski TF The host STING pathway at the interface of cancer and immunity. J Clin Invest (2016) 126(7):2404–11. 10.1172/JCI86892 PMC4922692 27367184

[93] Yan N Immune Diseases Associated with TREX1 and STING Dysfunction. J Interferon Cytokine Res (2017) 37(5):198–206. 10.1089/jir.2016.0086 28475463 PMC5439420

[94] Bader V Winklhofer KF Mitochondria at the interface between neurodegeneration and neuroinflammation. Semin Cell Dev Biol (2020) 99:163–71. 10.1016/j.semcdb.2019.05.028 31154011

[95] Li T Chen ZJ The cGAS-cGAMP-STING pathway connects DNA damage to inflammation, senescence, and cancer. J Exp Med (2018) 215(5):1287–99. 10.1084/jem.20180139 PMC5940270 29622565

[96] Moltedo O Remondelli P Amodio G The Mitochondria-Endoplasmic Reticulum Contacts and Their Critical Role in Aging and Age-Associated Diseases. Front Cell Dev Biol (2019) 7:172. 10.3389/fcell.2019.00172 31497601 PMC6712070

[97] Bai J Liu F The cGAS-cGAMP-STING Pathway: A Molecular Link Between Immunity and Metabolism. Diabetes (2019) 68(6):1099–108. 10.2337/dbi18-0052 PMC6610018 31109939

[98] King KR Aguirre AD Ye YX Sun Y Roh JD Ng RP Jr. IRF3 and type I interferons fuel a fatal response to myocardial infarction. Nat Med (2017) 23(12):1481–7. 10.1038/nm.4428 PMC6477926 29106401

[99] Deng Z Chong Z Law CS Mukai K Ho FO Martinu T A defect in COPI-mediated transport of STING causes immune dysregulation in COPA syndrome. J Exp Med (2020) 217(11):e20201045. 10.1084/jem.20201045 32725126 PMC7596814

[100] An J Durcan L Karr RM Briggs TA Rice GI Teal TH Expression of Cyclic GMP-AMP Synthase in Patients With Systemic Lupus Erythematosus. Arthritis Rheumatol (2017) 69(4):800–7. 10.1002/art.40002 27863149

[101] Bennett L Palucka AK Arce E Cantrell V Borvak J Banchereau J Interferon and granulopoiesis signatures in systemic lupus erythematosus blood. J Exp Med (2003) 197(6):711–23. 10.1084/jem.20021553 PMC2193846 12642603

[102] Sharma S Campbell AM Chan J Schattgen SA Orlowski GM Nayar R Suppression of systemic autoimmunity by the innate immune adaptor STING. Proc Natl Acad Sci U S A (2015) 112(7):E710–7. 10.1073/pnas.1420217112 PMC4343138 25646421

[103] Pestal K Funk CC Snyder JM Price ND Treuting PM Stetson DB Isoforms of RNA-Editing Enzyme ADAR1 Independently Control Nucleic Acid Sensor MDA5-Driven Autoimmunity and Multi-organ Development. Immunity (2015) 43(5):933–44. 10.1016/j.immuni.2015.11.001 PMC4654992 26588779

[104] Deng L Liang H Xu M Yang X Burnette B Arina A STING-Dependent Cytosolic DNA Sensing Promotes Radiation-Induced Type I Interferon-Dependent Antitumor Immunity in Immunogenic Tumors. Immunity (2014) 41(5):843–52. 10.1016/j.immuni.2014.10.019 PMC5155593 25517616

[105] Fuertes MB Kacha AK Kline J Woo SR Kranz DM Murphy KM Host type I IFN signals are required for antitumor CD8+ T cell responses through CD8{alpha}+ dendritic cells. J Exp Med (2011) 208(10):2005–16. 10.1084/jem.20101159 PMC3182064 21930765

[106] Flood BA Higgs EF Li S Luke JJ Gajewski TF STING pathway agonism as a cancer therapeutic. Immunol Rev (2019) 290(1):24–38. 10.1111/imr.12765 31355488 PMC6814203

[107] Huang L Li L Lemos H Chandler PR Pacholczyk G Baban B Cutting edge: DNA sensing via the STING adaptor in myeloid dendritic cells induces potent tolerogenic responses. J Immunol (2013) 191(7):3509–13. 10.4049/jimmunol.1301419 PMC3788571 23986532

[108] Lemos H Mohamed E Huang L Ou R Pacholczyk G Arbab AS STING Promotes the Growth of Tumors Characterized by Low Antigenicity via IDO Activation. Cancer Res (2016) 76(8):2076–81. 10.1158/0008-5472.CAN-15-1456 PMC4873329 26964621

[109] Chen Q Boire A Jin X Valiente M Er EE Lopez-Soto A Carcinoma-astrocyte gap junctions promote brain metastasis by cGAMP transfer. Nature (2016) 533(7604):493–8. 10.1038/nature18268 PMC5021195 27225120

[110] Cao DJ Schiattarella GG Villalobos E Jiang N May HI Li T Cytosolic DNA Sensing Promotes Macrophage Transformation and Governs Myocardial Ischemic Injury. Circulation (2018) 137(24):2613–34. 10.1161/CIRCULATIONAHA.117.031046 PMC5997506 29437120

[111] Lemos H Mohamed E Huang L Chandler PR Ou R Pacholczyk R Stimulator of interferon genes agonists attenuate type I diabetes progression in NOD mice. Immunology (2019) 158(4):353–61. 10.1111/imm.13122 PMC6856934 31557322

[112] Hu HQ Qiao JT Liu FQ Wang JB Sha S He Q The STING-IRF3 pathway is involved in lipotoxic injury of pancreatic beta cells in type 2 diabetes. Mol Cell Endocrinol (2020) 518:110890. 10.1016/j.mce.2020.110890 32781250

[113] Diamond JM Vanpouille-Box C Spada S Rudqvist NP Chapman JR Ueberheide BM Exosomes Shuttle TREX1-Sensitive IFN-Stimulatory dsDNA from Irradiated Cancer Cells to DCs. Cancer Immunol Res (2018) 6(8):910–20. 10.1158/2326-6066.CIR-17-0581 PMC6072562 29907693

[114] Torralba D Baixauli F Villarroya-Beltri C Fernandez-Delgado I Latorre-Pellicer A Acin-Perez R Priming of dendritic cells by DNA-containing extracellular vesicles from activated T cells through antigen-driven contacts. Nat Commun (2018) 9(1):2658. 10.1038/s41467-018-05077-9 29985392 PMC6037695

[115] Lan YY Londono D Bouley R Rooney MS Hacohen N Dnase2a deficiency uncovers lysosomal clearance of damaged nuclear DNA via autophagy. Cell Rep (2014) 9(1):180–92. 10.1016/j.celrep.2014.08.074 PMC4555847 25284779

[116] Li L Yin Q Kuss P Maliga Z Millan JL Wu H Hydrolysis of 2’3’-cGAMP by ENPP1 and design of nonhydrolyzable analogs. Nat Chem Biol (2014) 10(12):1043–8. 10.1038/nchembio.1661 PMC4232468 25344812

[117] Mackenzie KJ Carroll P Martin CA Murina O Fluteau A Simpson DJ cGAS surveillance of micronuclei links genome instability to innate immunity. Nature (2017) 548(7668):461–5. 10.1038/nature23449 PMC5870830 28738408

[118] Harding SM Benci JL Irianto J Discher DE Minn AJ Greenberg RA Mitotic progression following DNA damage enables pattern recognition within micronuclei. Nature (2017) 548(7668):466–70. 10.1038/nature23470 PMC5857357 28759889

[119] Ablasser A Schmid-Burgk JL Hemmerling I Horvath GL Schmidt T Latz E Cell intrinsic immunity spreads to bystander cells via the intercellular transfer of cGAMP. Nature (2013) 503(7477):530–4. 10.1038/nature12640 PMC4142317 24077100

[120] Marcus A Mao AJ Lensink-Vasan M Wang L Vance RE Raulet DH Tumor-Derived cGAMP Triggers a STING-Mediated Interferon Response in Non-tumor Cells to Activate the NK Cell Response. Immunity (2018) 49(4):754–63.e4. 10.1016/j.immuni.2018.09.016 30332631 PMC6488306

[121] Ku JWK Chen Y Lim BJW Gasser S Crasta KC Gan YH Bacterial-induced cell fusion is a danger signal triggering cGAS-STING pathway via micronuclei formation. Proc Natl Acad Sci U S A (2020) 117(27):15923–34. 10.1073/pnas.2006908117 PMC7355030 32571920

[122] Sharma M Rajendrarao S Shahani N Ramirez-Jarquin UN Subramaniam S Cyclic GMP-AMP synthase promotes the inflammatory and autophagy responses in Huntington disease. Proc Natl Acad Sci U S A (2020) 117(27):15989–99. 10.1073/pnas.2002144117 PMC7354937 32581130

[123] Crow YJ Hayward BE Parmar R Robins P Leitch A Ali M Mutations in the gene encoding the 3’-5’ DNA exonuclease TREX1 cause Aicardi-Goutieres syndrome at the AGS1 locus. Nat Genet (2006) 38(8):917–20. 10.1038/ng1845 16845398

[124] Stetson DB Ko JS Heidmann T Medzhitov R Trex1 prevents cell-intrinsic initiation of autoimmunity. Cell (2008) 134(4):587–98. 10.1016/j.cell.2008.06.032 PMC2626626 18724932

[125] Namjou B Kothari PH Kelly JA Glenn SB Ojwang JO Adler A Evaluation of the TREX1 gene in a large multi-ancestral lupus cohort. Genes Immun (2011) 12(4):270–9. 10.1038/gene.2010.73 PMC3107387 21270825

[126] Rice G Newman WG Dean J Patrick T Parmar R Flintoff K Heterozygous mutations in TREX1 cause familial chilblain lupus and dominant Aicardi-Goutieres syndrome. Am J Hum Genet (2007) 80(4):811–5. 10.1086/513443 PMC1852703 17357087

[127] Richards A van den Maagdenberg AM Jen JC Kavanagh D Bertram P Spitzer D C-terminal truncations in human 3’-5’ DNA exonuclease TREX1 cause autosomal dominant retinal vasculopathy with cerebral leukodystrophy. Nat Genet (2007) 39(9):1068–70. 10.1038/ng2082 17660820

[128] Crow YJ Leitch A Hayward BE Garner A Parmar R Griffith E Mutations in genes encoding ribonuclease H2 subunits cause Aicardi-Goutieres syndrome and mimic congenital viral brain infection. Nat Genet (2006) 38(8):910–6. 10.1038/ng1842 16845400

[129] Rice GI Bond J Asipu A Brunette RL Manfield IW Carr IM Mutations involved in Aicardi-Goutieres syndrome implicate SAMHD1 as regulator of the innate immune response. Nat Genet (2009) 41(7):829–32. 10.1038/ng.373 PMC4154505 19525956

[130] Coquel F Silva MJ Techer H Zadorozhny K Sharma S Nieminuszczy J SAMHD1 acts at stalled replication forks to prevent interferon induction. Nature (2018) 557(7703):57–61. 10.1038/s41586-018-0050-1 29670289

[131] Napirei M Karsunky H Zevnik B Stephan H Mannherz HG Moroy T Features of systemic lupus erythematosus in Dnase1-deficient mice. Nat Genet (2000) 25(2):177–81. 10.1038/76032 10835632

[132] Ahn J Gutman D Saijo S Barber GN STING manifests self DNA-dependent inflammatory disease. Proc Natl Acad Sci U S A (2012) 109(47):19386–91. 10.1073/pnas.1215006109 PMC3511090 23132945

[133] Yang YG Lindahl T Barnes DE Trex1 exonuclease degrades ssDNA to prevent chronic checkpoint activation and autoimmune disease. Cell (2007) 131(5):873–86. 10.1016/j.cell.2007.10.017 18045533

[134] Lim YW Sanz LA Xu X Hartono SR Chedin F Genome-wide DNA hypomethylation and RNA:DNA hybrid accumulation in Aicardi-Goutieres syndrome. eLife (2015) 4:e08007. 10.7554/eLife.08007 PMC4528086 26182405

[135] Hasan M Fermaintt CS Gao N Sakai T Miyazaki T Jiang S Cytosolic Nuclease TREX1 Regulates Oligosaccharyltransferase Activity Independent of Nuclease Activity to Suppress Immune Activation. Immunity (2015) 43(3):463–74. 10.1016/j.immuni.2015.07.022 PMC4575271 26320659

[136] Garcia-Romo GS Caielli S Vega B Connolly J Allantaz F Xu Z Netting neutrophils are major inducers of type I IFN production in pediatric systemic lupus erythematosus. Sci Trans Med (2011) 3(73):73ra20. 10.1126/scitranslmed.3001201 PMC3143837 21389264

[137] Wang H Li T Chen S Gu Y Ye S Neutrophil Extracellular Trap Mitochondrial DNA and Its Autoantibody in Systemic Lupus Erythematosus and a Proof-of-Concept Trial of Metformin. Arthritis Rheumatol (2015) 67(12):3190–200. 10.1002/art.39296 26245802

[138] Gehrke N Mertens C Zillinger T Wenzel J Bald T Zahn S Oxidative damage of DNA confers resistance to cytosolic nuclease TREX1 degradation and potentiates STING-dependent immune sensing. Immunity (2013) 39(3):482–95. 10.1016/j.immuni.2013.08.004 23993650

[139] Caielli S Athale S Domic B Murat E Chandra M Banchereau R Oxidized mitochondrial nucleoids released by neutrophils drive type I interferon production in human lupus. J Exp Med (2016) 213(5):697–713. 10.1084/jem.20151876 27091841 PMC4854735

[140] Lood C Blanco LP Purmalek MM Carmona-Rivera C De Ravin SS Smith CK Neutrophil extracellular traps enriched in oxidized mitochondrial DNA are interferogenic and contribute to lupus-like disease. Nat Med (2016) 22(2):146–53. 10.1038/nm.4027 PMC4742415 26779811

[141] Maekawa H Inoue T Ouchi H Jao TM Inoue R Nishi H Mitochondrial Damage Causes Inflammation via cGAS-STING Signaling in Acute Kidney Injury. Cell Rep (2019) 29(5):1261–73.e6. 10.1016/j.celrep.2019.09.050 31665638

[142] Fang C Mo F Liu L Du J Luo M Men K Oxidized mitochondrial DNA sensing by STING signaling promotes the antitumor effect of an irradiated immunogenic cancer cell vaccine. Cell Mol Immunol (2020). 10.1038/s41423-020-0456-1 PMC8429462 32398808

[143] Catteau A Roue G Yuste VJ Susin SA Despres P Expression of dengue ApoptoM sequence results in disruption of mitochondrial potential and caspase activation. Biochimie (2003) 85(8):789–93. 10.1016/S0300-9084(03)00139-1 14585546

[144] Yu CY Liang JJ Li JK Lee YL Chang BL Su CI Dengue Virus Impairs Mitochondrial Fusion by Cleaving Mitofusins. PLoS Pathog (2015) 11(12):e1005350. 10.1371/journal.ppat.1005350 26717518 PMC4696832

[145] West AP Khoury-Hanold W Staron M Tal MC Pineda CM Lang SM Mitochondrial DNA stress primes the antiviral innate immune response. Nature (2015) 520(7548):553–7. 10.1038/nature14156 PMC4409480 25642965

[146] Aguirre S Maestre AM Pagni S Patel JR Savage T Gutman D DENV inhibits type I IFN production in infected cells by cleaving human STING. PLoS Pathog (2012) 8(10):e1002934. 10.1371/journal.ppat.1002934 23055924 PMC3464218

[147] Moriyama M Koshiba T Ichinohe T Influenza A virus M2 protein triggers mitochondrial DNA-mediated antiviral immune responses. Nat Commun (2019) 10(1):4624. 10.1038/s41467-019-12632-5 31604929 PMC6789137

[148] Wiens KE Ernst JD The Mechanism for Type I Interferon Induction by Mycobacterium tuberculosis is Bacterial Strain-Dependent. PLoS Pathogens (2016) 12(8):e1005809. 10.1371/journal.ppat.1005809 27500737 PMC4976988

[149] Watson RO Bell SL MacDuff DA Kimmey JM Diner EJ Olivas J The Cytosolic Sensor cGAS Detects Mycobacterium tuberculosis DNA to Induce Type I Interferons and Activate Autophagy. Cell Host Microbe (2015) 17(6):811–9. 10.1016/j.chom.2015.05.004 PMC4466081 26048136

[150] Kim BR Kim BJ Kook YH Kim BJ Mycobacterium abscessus infection leads to enhanced production of type 1 interferon and NLRP3 inflammasome activation in murine macrophages via mitochondrial oxidative stress. PLoS Pathogens (2020) 16(3):e1008294. 10.1371/journal.ppat.1008294 32210476 PMC7094820

[151] Hetz C Zhang K Kaufman RJ Mechanisms, regulation and functions of the unfolded protein response. Nat Rev Mol Cell Biol (2020) 21(8):421–38. 10.1038/s41580-020-0250-z PMC8867924 32457508

[152] Ron D Walter P Signal integration in the endoplasmic reticulum unfolded protein response. Nat Rev Mol Cell Biol (2007) 8(7):519–29. 10.1038/nrm2199 17565364

[153] Smith JA Regulation of Cytokine Production by the Unfolded Protein Response; Implications for Infection and Autoimmunity. Front Immunol (2018) 9:422. 10.3389/fimmu.2018.00422 29556237 PMC5844972

[154] Grootjans J Kaser A Kaufman RJ Blumberg RS The unfolded protein response in immunity and inflammation. Nat Rev Immunol (2016) 16(8):469–84. 10.1038/nri.2016.62 PMC5310224 27346803

[155] Todd DJ Lee AH Glimcher LH The endoplasmic reticulum stress response in immunity and autoimmunity. Nat Rev Immunol (2008) 8(9):663–74. 10.1038/nri2359 18670423

[156] Martinon F Chen X Lee AH Glimcher LH TLR activation of the transcription factor XBP1 regulates innate immune responses in macrophages. Nat Immunol (2010) 11(5):411–8. 10.1038/ni.1857 PMC3113706 20351694

[157] Smith JA Turner MJ DeLay ML Klenk EI Sowders DP Colbert RA Endoplasmic reticulum stress and the unfolded protein response are linked to synergistic IFN-beta induction via X-box binding protein 1. Eur J Immunol (2008) 38(5):1194–203. 10.1002/eji.200737882 PMC2838478 18412159

[158] Liu YP Zeng L Tian A Bomkamp A Rivera D Gutman D Endoplasmic Reticulum Stress Regulates the Innate Immunity Critical Transcription Factor IRF3. J Immunol (2012) 189(9):4630–9. 10.4049/jimmunol.1102737 PMC3478468 23028052

[159] Sehgal P Szalai P Olesen C Praetorius HA Nissen P Christensen SB Inhibition of the sarco/endoplasmic reticulum (ER) Ca(2+)-ATPase by thapsigargin analogs induces cell death via ER Ca(2+) depletion and the unfolded protein response. J Biol Chem (2017) 292(48):19656–73. 10.1074/jbc.M117.796920 PMC5712609 28972171

[160] Larsen GA Skjellegrind HK Moe MC Vinje ML Berg-Johnsen J Endoplasmic reticulum dysfunction and Ca2+ deregulation in isolated CA1 neurons during oxygen and glucose deprivation. Neurochem Res (2005) 30(5):651–9. 10.1007/s11064-005-2753-6 16176069

[161] Toyoda H Kawano T Sato H Kato T Cellular mechanisms underlying the rapid depolarization caused by oxygen and glucose deprivation in layer III pyramidal cells of the somatosensory cortex. Neurosci Res (2020) S0168–0102(19):30682–0. 10.1016/j.neures.2020.03.003 32171781

[162] Petrasek J Iracheta-Vellve A Csak T Satishchandran A Kodys K Kurt-Jones EA STING-IRF3 pathway links endoplasmic reticulum stress with hepatocyte apoptosis in early alcoholic liver disease. Proc Natl Acad Sci U S A (2013) 110(41):16544–9. 10.1073/pnas.1308331110 PMC3799324 24052526

[163] Sen T Saha P Gupta R Foley LM Jiang T Abakumova OS Aberrant ER Stress Induced Neuronal-IFNbeta Elicits White Matter Injury Due to Microglial Activation and T-Cell Infiltration after TBI. J Neurosci (2020) 40(2):424–46. 10.1523/JNEUROSCI.0718-19.2019 PMC6948950 31694961

[164] Cui Y Zhao D Sreevatsan S Liu C Yang W Song Z Mycobacterium bovis Induces Endoplasmic Reticulum Stress Mediated-Apoptosis by Activating IRF3 in a Murine Macrophage Cell Line. Front Cell Infect Microbiol (2016) 6:182. 10.3389/fcimb.2016.00182 28018864 PMC5149527

[165] Guimaraes ES Gomes MTR Campos PC Mansur DS Dos Santos AA Harms J Brucella abortus Cyclic Dinucleotides Trigger STING-Dependent Unfolded Protein Response That Favors Bacterial Replication. J Immunol (2019) 202(9):2671–81. 10.4049/jimmunol.1801233 PMC6478548 30894428

[166] Zhang Y Chen W Wang Y STING is an essential regulator of heart inflammation and fibrosis in mice with pathological cardiac hypertrophy via endoplasmic reticulum (ER) stress. BioMed Pharmacother (2020) 125:110022. 10.1016/j.biopha.2020.110022 32106379

[167] Moretti J Roy S Bozec D Martinez J Chapman JR Ueberheide B STING Senses Microbial Viability to Orchestrate Stress-Mediated Autophagy of the Endoplasmic Reticulum. Cell (2017) 171(4):809–23.e13. 10.1016/j.cell.2017.09.034 29056340 PMC5811766

[168] Wu J Chen YJ Dobbs N Sakai T Liou J Miner JJ STING-mediated disruption of calcium homeostasis chronically activates ER stress and primes T cell death. J Exp Med (2019) 216(4):867–83. 10.1084/jem.20182192 PMC6446864 30886058

[169] Liu Y Jesus AA Marrero B Yang D Ramsey SE Sanchez GAM Activated STING in a vascular and pulmonary syndrome. N Engl J Med (2014) 371(6):507–18. 10.1056/NEJMoa1312625 PMC4174543 25029335

[170] Warner JD Irizarry-Caro RA Bennion BG Ai TL Smith AM Miner CA STING-associated vasculopathy develops independently of IRF3 in mice. J Exp Med (2017) 214(11):3279–92. 10.1084/jem.20171351 PMC5679177 28951494

[171] Gorlach A Bertram K Hudecova S Krizanova O Calcium and ROS: A mutual interplay. Redox Biol (2015) 6:260–71. 10.1016/j.redox.2015.08.010 PMC4556774 26296072

[172] Berridge MJ Lipp P Bootman MD The versatility and universality of calcium signalling. Nat Rev Mol Cell Biol (2000) 1(1):11–21. 10.1038/35036035 11413485

[173] Kaufman RJ Malhotra JD Calcium trafficking integrates endoplasmic reticulum function with mitochondrial bioenergetics. Biochim Biophys Acta (2014) 1843(10):2233–9. 10.1016/j.bbamcr.2014.03.022 PMC4285153 24690484

[174] Zapun A Darby NJ Tessier DC Michalak M Bergeron JJ Thomas DY Enhanced catalysis of ribonuclease B folding by the interaction of calnexin or calreticulin with ERp57. J Biol Chem (1998) 273(11):6009–12. 10.1074/jbc.273.11.6009 9497314

[175] Hebert DN Molinari M Flagging and docking: dual roles for N-glycans in protein quality control and cellular proteostasis. Trends Biochem Sci (2012) 37(10):404–10. 10.1016/j.tibs.2012.07.005 PMC3459134 22921611

[176] Hendershot LM The ER function BiP is a master regulator of ER function. Mt Sinai J Med (2004) 71(5):289–97. 15543429

[177] Vandecaetsbeek I Vangheluwe P Raeymaekers L Wuytack F Vanoevelen J The Ca2+ pumps of the endoplasmic reticulum and Golgi apparatus. Cold Spring Harb Perspect Biol (2011) 3(5):a004184. 10.1101/cshperspect.a004184 21441596 PMC3101839

[178] Mendes CC Gomes DA Thompson M Souto NC Goes TS Goes AM The type III inositol 1,4,5-trisphosphate receptor preferentially transmits apoptotic Ca2+ signals into mitochondria. J Biol Chem (2005) 280(49):40892–900. 10.1074/jbc.M506623200 16192275

[179] Kiviluoto S Vervliet T Ivanova H Decuypere JP De Smedt H Missiaen L Regulation of inositol 1,4,5-trisphosphate receptors during endoplasmic reticulum stress. Biochim Biophys Acta (2013) 1833(7):1612–24. 10.1016/j.bbamcr.2013.01.026 23380704

[180] Gudlur A Zeraik AE Hirve N Rajanikanth V Bobkov AA Ma G Calcium sensing by the STIM1 ER-luminal domain. Nat Commun (2018) 9(1):4536. 10.1038/s41467-018-06816-8 30382093 PMC6208404

[181] Park CY Hoover PJ Mullins FM Bachhawat P Covington ED Raunser S STIM1 clusters and activates CRAC channels via direct binding of a cytosolic domain to Orai1. Cell (2009) 136(5):876–90. 10.1016/j.cell.2009.02.014 PMC2670439 19249086

[182] Shu C Yi G Watts T Kao CC Li P Structure of STING bound to cyclic di-GMP reveals the mechanism of cyclic dinucleotide recognition by the immune system. Nat Struct Mol Biol (2012) 19(7):722–4. 10.1038/nsmb.2331 PMC3392545 22728658

[183] Zhang W Zhou Q Xu W Cai Y Yin Z Gao X DNA-dependent activator of interferon-regulatory factors (DAI) promotes lupus nephritis by activating the calcium pathway. J Biol Chem (2013) 288(19):13534–50. 10.1074/jbc.M113.457218 PMC3650390 23553627

[184] Gamage AM Lee KO Gan YH Anti-Cancer Drug HMBA Acts as an Adjuvant during Intracellular Bacterial Infections by Inducing Type I IFN through STING. J Immunol (2017) 199(7):2491–502. 10.4049/jimmunol.1602162 28827286

[185] Hare DN Collins SE Mukherjee S Loo YM Gale M Jr. Janssen LJ Membrane Perturbation-Associated Ca2+ Signaling and Incoming Genome Sensing Are Required for the Host Response to Low-Level Enveloped Virus Particle Entry. J Virol (2015) 90(6):3018–27. 10.1128/JVI.02642-15 PMC4810640 26719279

[186] Hurley RL Anderson KA Franzone JM Kemp BE Means AR Witters LA The Ca2+/calmodulin-dependent protein kinase kinases are AMP-activated protein kinase kinases. J Biol Chem (2005) 280(32):29060–6. 10.1074/jbc.M503824200 15980064

[187] Kwon D Sesaki H Kang SJ Intracellular calcium is a rheostat for the STING signaling pathway. Biochem Biophys Res Commun (2018) 500(2):497–503. 10.1016/j.bbrc.2018.04.117 29673589

[188] Kyttaris VC Zhang Z Kampagianni O Tsokos GC Calcium signaling in systemic lupus erythematosus T cells: a treatment target. Arthritis Rheumatol (2011) 63(7):2058–66. 10.1002/art.30353 PMC3128171 21437870

[189] Ichinose K Juang YT Crispin JC Kis-Toth K Tsokos GC Suppression of autoimmunity and organ pathology in lupus-prone mice upon inhibition of calcium/calmodulin-dependent protein kinase type IV. Arthritis Rheumatol (2011) 63(2):523–9. 10.1002/art.30085 PMC3030625 20954187

[190] Koga T Ichinose K Mizui M Crispin JC Tsokos GC Calcium/calmodulin-dependent protein kinase IV suppresses IL-2 production and regulatory T cell activity in lupus. J Immunol (2012) 189(7):3490–6. 10.4049/jimmunol.1201785 PMC3448834 22942433

[191] Srikanth S Woo JS Wu B El-Sherbiny YM Leung J Chupradit K The Ca(2+) sensor STIM1 regulates the type I interferon response by retaining the signaling adaptor STING at the endoplasmic reticulum. Nat Immunol (2019) 20(2):152–62. 10.1038/s41590-018-0287-8 PMC6340781 30643259

[192] Mathavarajah S Salsman J Dellaire G An emerging role for calcium signalling in innate and autoimmunity via the cGAS-STING axis. Cytokine Growth Factor Rev (2019) 50:43–51. 10.1016/j.cytogfr.2019.04.003 30955997

[193] Zhang H Zeng L Xie M Liu J Zhou B Wu R TMEM173 Drives Lethal Coagulation in Sepsis. Cell Host Microbe (2020) 27(4):556–70. 10.1016/j.chom.2020.02.004 PMC7316085 32142632

[194] Marchi S Patergnani S Pinton P The endoplasmic reticulum-mitochondria connection: one touch, multiple functions. Biochim Biophys Acta (2014) 1837(4):461–9. 10.1016/j.bbabio.2013.10.015 24211533

[195] Flis VV Daum G Lipid transport between the endoplasmic reticulum and mitochondria. Cold Spring Harb Perspect Biol (2013) 5(6):a013235. 10.1101/cshperspect.a013235 23732475 PMC3660828

[196] Friedman JR Lackner LL West M DiBenedetto JR Nunnari J Voeltz GK ER tubules mark sites of mitochondrial division. Science (2011) 334(6054):358–62. 10.1126/science.1207385 PMC3366560 21885730

[197] Chakrabarti R Ji WK Stan RV de Juan Sanz J Ryan TA Higgs HN INF2-mediated actin polymerization at the ER stimulates mitochondrial calcium uptake, inner membrane constriction, and division. J Cell Biol (2018) 217(1):251–68. 10.1083/jcb.201709111 PMC5748994 29142021

[198] de Brito OM Scorrano L Mitofusin 2 tethers endoplasmic reticulum to mitochondria. Nature (2008) 456(7222):605–10. 10.1038/nature07534 19052620

[199] Gomez-Suaga P Paillusson S Stoica R Noble W Hanger DP Miller CCJ The ER-Mitochondria Tethering Complex VAPB-PTPIP51 Regulates Autophagy. Curr Biol (2017) 27(3):371–85. 10.1016/j.cub.2016.12.038 PMC5300905 28132811

[200] Szabadkai G Bianchi K Varnai P De Stefani D Wieckowski MR Cavagna D Chaperone-mediated coupling of endoplasmic reticulum and mitochondrial Ca2+ channels. J Cell Biol (2006) 175(6):901–11. 10.1083/jcb.200608073 PMC2064700 17178908

[201] De Stefani D Raffaello A Teardo E Szabo I Rizzuto R A forty-kilodalton protein of the inner membrane is the mitochondrial calcium uniporter. Nature (2011) 476(7360):336–40. 10.1038/nature10230 PMC4141877 21685888

[202] Hayashi T Su TP Sigma-1 receptor chaperones at the ER-mitochondrion interface regulate Ca(2+) signaling and cell survival. Cell (2007) 131(3):596–610. 10.1016/j.cell.2007.08.036 17981125

[203] Simmen T Lynes EM Gesson K Thomas G Oxidative protein folding in the endoplasmic reticulum: tight links to the mitochondria-associated membrane (MAM). Biochim Biophys Acta (2010) 1798(8):1465–73. 10.1016/j.bbamem.2010.04.009 PMC2885528 20430008

[204] Morgan MJ Liu ZG Crosstalk of reactive oxygen species and NF-kappaB signaling. Cell Res (2011) 21(1):103–15. 10.1038/cr.2010.178 PMC3193400 21187859

[205] Dupre-Crochet S Erard M Nubetae O ROS production in phagocytes: why, when, and where? J Leukoc Biol (2013) 94(4):657–70. 10.1189/jlb.1012544 23610146

[206] Harijith A Ebenezer DL Natarajan V Reactive oxygen species at the crossroads of inflammasome and inflammation. Front Physiol (2014) 5:352. 10.3389/fphys.2014.00352 25324778 PMC4179323

[207] Tu BP Weissman JS Oxidative protein folding in eukaryotes: mechanisms and consequences. J Cell Biol (2004) 164(3):341–6. 10.1083/jcb.200311055 PMC2172237 14757749

[208] Kozlov G Maattanen P Thomas DY Gehring K A structural overview of the PDI family of proteins. FEBS J (2010) 277(19):3924–36. 10.1111/j.1742-4658.2010.07793.x 20796029

[209] Frand AR Kaiser CA The ERO1 gene of yeast is required for oxidation of protein dithiols in the endoplasmic reticulum. Mol Cell (1998) 1(2):161–70. 10.1016/S1097-2765(00)80017-9 9659913

[210] Pollard MG Travers KJ Weissman JS Ero1p: a novel and ubiquitous protein with an essential role in oxidative protein folding in the endoplasmic reticulum. Mol Cell (1998) 1(2):171–82. 10.1016/S1097-2765(00)80018-0 9659914

[211] Marciniak SJ Yun CY Oyadomari S Novoa I Zhang Y Jungreis R CHOP induces death by promoting protein synthesis and oxidation in the stressed endoplasmic reticulum. Genes Dev (2004) 18(24):3066–77. 10.1101/gad.1250704 PMC535917 15601821

[212] Hansen HG Schmidt JD Soltoft CL Ramming T Geertz-Hansen HM Christensen B Hyperactivity of the Ero1alpha oxidase elicits endoplasmic reticulum stress but no broad antioxidant response. J Biol Chem (2012) 287(47):39513–23. 10.1074/jbc.M112.405050 PMC3501080 23027870

[213] Laurindo FR Araujo TL Abrahao TB Nox NADPH oxidases and the endoplasmic reticulum. Antioxid Redox Signaling (2014) 20(17):2755–75. 10.1089/ars.2013.5605 PMC4026305 24386930

[214] Touyz RM Anagnostopoulou A Rios F Montezano AC Camargo LL NOX5: Molecular biology and pathophysiology. Exp Physiol (2019) 104(5):605–16. 10.1113/EP086204 PMC6519284 30801870

[215] Cao SS Kaufman RJ Endoplasmic reticulum stress and oxidative stress in cell fate decision and human disease. Antioxid Redox Signaling (2014) 21(3):396–413. 10.1089/ars.2014.5851 PMC4076992 24702237

[216] Forrester SJ Kikuchi DS Hernandes MS Xu Q Griendling KK Reactive Oxygen Species in Metabolic and Inflammatory Signaling. Circ Res (2018) 122(6):877–902. 10.1161/CIRCRESAHA.117.311401 29700084 PMC5926825

[217] Wei PC Hsieh YH Su MI Jiang X Hsu PH Lo WT Loss of the oxidative stress sensor NPGPx compromises GRP78 chaperone activity and induces systemic disease. Mol Cell (2012) 48(5):747–59. 10.1016/j.molcel.2012.10.007 PMC3582359 23123197

[218] Hwang C Sinskey AJ Lodish HF Oxidized redox state of glutathione in the endoplasmic reticulum. Science (1992) 257(5076):1496–502. 10.1126/science.1523409 1523409

[219] Balaban RS Nemoto S Finkel T Mitochondria, oxidants, and aging. Cell (2005) 120(4):483–95. 10.1016/j.cell.2005.02.001 15734681

[220] Shanmugasundaram K Nayak BK Friedrichs WE Kaushik D Rodriguez R Block K NOX4 functions as a mitochondrial energetic sensor coupling cancer metabolic reprogramming to drug resistance. Nat Commun (2017) 8(1):997. 10.1038/s41467-017-01106-1 29051480 PMC5648812

[221] Zhang H Bosch-Marce M Shimoda LA Tan YS Baek JH Wesley JB Mitochondrial autophagy is an HIF-1-dependent adaptive metabolic response to hypoxia. J Biol Chem (2008) 283(16):10892–903. 10.1074/jbc.M800102200 PMC2447655 18281291

[222] Kim JW Tchernyshyov I Semenza GL Dang CV HIF-1-mediated expression of pyruvate dehydrogenase kinase: a metabolic switch required for cellular adaptation to hypoxia. Cell Metab (2006) 3(3):177–85. 10.1016/j.cmet.2006.02.002 16517405

[223] Hwang HJ Lynn SG Vengellur A Saini Y Grier EA Ferguson-Miller SM Hypoxia Inducible Factors Modulate Mitochondrial Oxygen Consumption and Transcriptional Regulation of Nuclear-Encoded Electron Transport Chain Genes. Biochemistry (2015) 54(24):3739–48. 10.1021/bi5012892 PMC5957085 26030260

[224] Qutub AA Popel AS Reactive oxygen species regulate hypoxia-inducible factor 1alpha differentially in cancer and ischemia. Mol Cell Biol (2008) 28(16):5106–19. 10.1128/MCB.00060-08 PMC2519710 18559422

[225] Norton M Ng AC Baird S Dumoulin A Shutt T Mah N ROMO1 is an essential redox-dependent regulator of mitochondrial dynamics. Sci Signal (2014) 7(310):ra10. 10.1126/scisignal.2004374 24473195

[226] Wu S Zhou F Zhang Z Xing D Mitochondrial oxidative stress causes mitochondrial fragmentation via differential modulation of mitochondrial fission-fusion proteins. FEBS J (2011) 278(6):941–54. 10.1111/j.1742-4658.2011.08010.x 21232014

[227] Yu T Robotham JL Yoon Y Increased production of reactive oxygen species in hyperglycemic conditions requires dynamic change of mitochondrial morphology. Proc Natl Acad Sci U S A (2006) 103(8):2653–8. 10.1073/pnas.0511154103 PMC1413838 16477035

[228] Cardenas C Miller RA Smith I Bui T Molgo J Muller M Essential regulation of cell bioenergetics by constitutive InsP3 receptor Ca2+ transfer to mitochondria. Cell (2010) 142(2):270–83. 10.1016/j.cell.2010.06.007 PMC2911450 20655468

[229] Booth DM Enyedi B Geiszt M Varnai P Hajnoczky G Redox Nanodomains Are Induced by and Control Calcium Signaling at the ER-Mitochondrial Interface. Mol Cell (2016) 63(2):240–8. 10.1016/j.molcel.2016.05.040 PMC4998968 27397688

[230] Andersson DC Betzenhauser MJ Reiken S Meli AC Umanskaya A Xie W Ryanodine receptor oxidation causes intracellular calcium leak and muscle weakness in aging. Cell Metab (2011) 14(2):196–207. 10.1016/j.cmet.2011.05.014 21803290 PMC3690519

[231] Gilady SY Bui M Lynes EM Benson MD Watts R Vance JE Ero1alpha requires oxidizing and normoxic conditions to localize to the mitochondria-associated membrane (MAM). Cell Stress Chaperones (2010) 15(5):619–29. 10.1007/s12192-010-0174-1 PMC3006622 20186508

[232] Li G Mongillo M Chin KT Harding H Ron D Marks AR Role of ERO1-alpha-mediated stimulation of inositol 1,4,5-triphosphate receptor activity in endoplasmic reticulum stress-induced apoptosis. J Cell Biol (2009) 186(6):783–92. 10.1083/jcb.200904060 PMC2753154 19752026

[233] Anelli T Bergamelli L Margittai E Rimessi A Fagioli C Malgaroli A Ero1alpha regulates Ca(2+) fluxes at the endoplasmic reticulum-mitochondria interface (MAM). Antioxid Redox Signaling (2012) 16(10):1077–87. 10.1089/ars.2011.4004 21854214

[234] Dikalov SI Nazarewicz RR Bikineyeva A Hilenski L Lassegue B Griendling KK Nox2-induced production of mitochondrial superoxide in angiotensin II-mediated endothelial oxidative stress and hypertension. Antioxid Redox Signaling (2014) 20(2):281–94. 10.1089/ars.2012.4918 PMC3887459 24053613

[235] Nazarewicz RR Dikalova AE Bikineyeva A Dikalov SI Nox2 as a potential target of mitochondrial superoxide and its role in endothelial oxidative stress. Am J Physiol Heart Circ Physiol (2013) 305(8):H1131–40. 10.1152/ajpheart.00063.2013 PMC3798790 23955717

[236] Hawkins BJ Irrinki KM Mallilankaraman K Lien YC Wang Y Bhanumathy CD S-glutathionylation activates STIM1 and alters mitochondrial homeostasis. J Cell Biol (2010) 190(3):391–405. 10.1083/jcb.201004152 20679432 PMC2922639

[237] Berna-Erro A Braun A Kraft R Kleinschnitz C Schuhmann MK Stegner D STIM2 regulates capacitive Ca2+ entry in neurons and plays a key role in hypoxic neuronal cell death. Sci Signal (2009) 2(93):ra67. 10.1126/scisignal.2000522 19843959

[238] Brechard S Melchior C Plancon S Schenten V Tschirhart EJ Store-operated Ca2+ channels formed by TRPC1, TRPC6 and Orai1 and non-store-operated channels formed by TRPC3 are involved in the regulation of NADPH oxidase in HL-60 granulocytes. Cell Calcium (2008) 44(5):492–506. 10.1016/j.ceca.2008.03.002 18436303

[239] Gandhirajan RK Meng S Chandramoorthy HC Mallilankaraman K Mancarella S Gao H Blockade of NOX2 and STIM1 signaling limits lipopolysaccharide-induced vascular inflammation. J Clin Invest (2013) 123(2):887–902. 10.1172/JCI65647 23348743 PMC3561818

[240] Li Y Schwabe RF DeVries-Seimon T Yao PM Gerbod-Giannone MC Tall AR Free cholesterol-loaded macrophages are an abundant source of tumor necrosis factor-alpha and interleukin-6: model of NF-kappaB- and map kinase-dependent inflammation in advanced atherosclerosis. J Biol Chem (2005) 280(23):21763–72. 10.1074/jbc.M501759200 15826936

[241] Mori T Hayashi T Hayashi E Su TP Sigma-1 receptor chaperone at the ER-mitochondrion interface mediates the mitochondrion-ER-nucleus signaling for cellular survival. PLoS One (2013) 8(10):e76941. 10.1371/journal.pone.0076941 24204710 PMC3799859

[242] Verfaillie T Rubio N Garg AD Bultynck G Rizzuto R Decuypere JP PERK is required at the ER-mitochondrial contact sites to convey apoptosis after ROS-based ER stress. Cell Death Differ (2012) 19(11):1880–91. 10.1038/cdd.2012.74 PMC3469056 22705852

[243] Cullinan SB Zhang D Hannink M Arvisais E Kaufman RJ Diehl JA Nrf2 is a direct PERK substrate and effector of PERK-dependent cell survival. Mol Cell Biol (2003) 23(20):7198–209. 10.1128/MCB.23.20.7198-7209.2003 PMC230321 14517290

[244] Harding HP Zhang Y Bertolotti A Zeng H Ron D Perk is essential for translational regulation and cell survival during the unfolded protein response. Mol Cell (2000) 5(5):897–904. 10.1016/S1097-2765(00)80330-5 10882126

[245] Lee YR Kuo SH Lin CY Fu PJ Lin YS Yeh TM Dengue virus-induced ER stress is required for autophagy activation, viral replication, and pathogenesis both in vitro and in vivo. Sci Rep (2018) 8(1):489. 10.1038/s41598-017-18909-3 29323257 PMC5765116

[246] Yan M Shu S Guo C Tang C Dong Z Endoplasmic reticulum stress in ischemic and nephrotoxic acute kidney injury. Ann Med (2018) 50(5):381–90. 10.1080/07853890.2018.1489142 PMC6333465 29895209

[247] Hurst KE Lawrence KA Essman MT Walton ZJ Leddy LR Thaxton JE Endoplasmic Reticulum Stress Contributes to Mitochondrial Exhaustion of CD8(+) T Cells. Cancer Immunol Res (2019) 7(3):476–86. 10.1158/2326-6066.CIR-18-0182 PMC6397687 30659052

[248] Castro F Cardoso AP Goncalves RM Serre K Oliveira MJ Interferon-Gamma at the Crossroads of Tumor Immune Surveillance or Evasion. Front Immunol (2018) 9:847. 10.3389/fimmu.2018.00847 29780381 PMC5945880

[249] Cubillos-Ruiz JR Silberman PC Rutkowski MR Chopra S Perales-Puchalt A Song M ER Stress Sensor XBP1 Controls Anti-tumor Immunity by Disrupting Dendritic Cell Homeostasis. Cell (2015) 161(7):1527–38. 10.1016/j.cell.2015.05.025 PMC4580135 26073941

[250] Cubillos-Ruiz JR Bettigole SE Glimcher LH Tumorigenic and Immunosuppressive Effects of Endoplasmic Reticulum Stress in Cancer. Cell (2017) 168(4):692–706. 10.1016/j.cell.2016.12.004 28187289 PMC5333759

[251] Mohamed E Sierra RA Trillo-Tinoco J Cao Y Innamarato P Payne KK The Unfolded Protein Response Mediator PERK Governs Myeloid Cell-Driven Immunosuppression in Tumors through Inhibition of STING Signaling. Immunity (2020) 52(4):668–82.e7. 10.1016/j.immuni.2020.03.004 32294407 PMC7207019

[252] Olagnier D Brandtoft AM Gunderstofte C Villadsen NL Krapp C Thielke AL Nrf2 negatively regulates STING indicating a link between antiviral sensing and metabolic reprogramming. Nat Commun (2018) 9(1):3506. 10.1038/s41467-018-05861-7 30158636 PMC6115435

[253] Hetz C Papa FR The Unfolded Protein Response and Cell Fate Control. Mol Cell (2018) 69(2):169–81. 10.1016/j.molcel.2017.06.017 29107536

[254] Rongvaux A Jackson R Harman CC Li T West AP de Zoete MR Apoptotic caspases prevent the induction of type I interferons by mitochondrial DNA. Cell (2014) 159(7):1563–77. 10.1016/j.cell.2014.11.037 PMC4272443 25525875

[255] Kim J Gupta R Blanco LP Yang S Shteinfer-Kuzmine A Wang K VDAC oligomers form mitochondrial pores to release mtDNA fragments and promote lupus-like disease. Science (2019) 366(6472):1531–6. 10.1126/science.aav4011 PMC8325171 31857488

